# Nutrition‐Associated Biomarkers in Predicting Intravenous Immunoglobulin Resistance and Coronary Artery Lesions in Kawasaki Disease: A Systematic Review and Meta‐Analysis

**DOI:** 10.1002/fsn3.4647

**Published:** 2024-12-15

**Authors:** Ling Liu, Rui Chen, Hong Wang, Honglu Yu, Zeyu Ai, Xiaofei Zhang

**Affiliations:** ^1^ Department of Pediatrics China‐Japan Union Hospital of Jilin University Changchun Jilin China

**Keywords:** coronary artery lesions, intravenous immunoglobulin resistance, Kawasaki disease, nutrition

## Abstract

Several studies explored the associations of pre‐albumin (PA)/albumin (ALB) and ALB‐combined indicators (prognostic nutrition index [PNI], albumin‐to‐globulin ratio [AGR], bilirubin‐to‐albumin [BAR], and C‐reactive protein/albumin ratio [CAR]) with intravenous immunoglobulin (IVIG) resistance and coronary artery lesions (CALs) in Kawasaki disease (KD) patients. However, the results were controversial. A meta‐analysis was conducted to reconfirm their associations and predictive performance. Databases of PubMed, EMBASE, and the Cochrane library were searched. The pooled standardized mean difference (SMD) or odds ratios (ORs) assessed the association, while the pooled area under the receiver operating characteristic curve (AUC) evaluated the predictive power. Ninety‐four studies were included. Overall and subgroup meta‐analyses demonstrated lower ALB and higher CAR were associated with IVIG resistance (ALB: SMD = −0.61; OR = 0.83; CAR: SMD = 1.47; OR = 1.69) and CALs (ALB: SMD = −0.56; OR = 0.92; CAR: SMD = 0.52). PNI was reduced in IVIG‐resistant (SMD = −0.82) and coronary artery aneurysm (SMD = −0.18) patients in subgroup analysis and high PNI predicted the decreased risk of CALs in overall analysis (OR = 0.82). ALB, CAR, and PNI were a good or fair biomarker for differentiating IVIG‐resistant (CALs) from IVIG‐sensitive (non‐CALs) patients (AUC > 0.6 or > 0.7). PA (SMD = −0.72) and BAR (SMD = 1.10) were differential in IVIG‐resistant, but not in CAL patients compared with controls. AGR was not associated with CALs (*p* > 0.05). In conclusion, ALB, CAR, PNI, PA, and BAR may represent promising biomarkers for the prediction of IVIG resistance and CALs in KD patients.

## Graphical abstract

Pooling the data from 94 articles indicated lower ALB, PNI and higher CAR were associated with an increased risk of IVIG resistance and CALs in Kawasaki disease children, with the predictive power of > 0.6 or > 0.7. Reduced PA and elevated BAR were also found in IVIG‐resistant patients compared with controls.

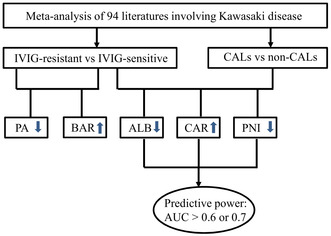

## 1 Introduction

Kawasaki disease (KD) is an acute self‐limited systemic vasculitis that predominantly affects children under the age of 5 years in East Asia (especially China, Korea and Japan) (Rowley and Shulman 2018) and manifests the clinical features of high fever, rash, cervical lymphadenopathy, conjunctivitis, chapped lips, swollen extremities, and skin sloughing (Lee et al. 2022). Coronary arteries are the most commonly damaged vessels and thus KD is often complicated by coronary artery lesions (CALs) (including dilatation [CAD] and/or aneurysms [CAA]) during the acute stage which subsequently may develop into myocardial ischemia, infarction, heart failure, and even sudden death due to lumen stenosis or occlusion (Ayusawa et al. 2022). This makes KD as a leading cause of acquired pediatric heart disease worldwide. Currently, high‐dose intravenous immunoglobulin (IVIG) (2 g/kg) combined with aspirin is considered as the first‐line treatment for KD; this regimen had been reported to alleviate symptoms and reduce the incidence of life‐threatening cardiac sequelae (Michihata and Suzuki 2022). However, approximately 30% of the patients still failed to respond to the IVIG therapy and exhibited persistent or recurrent fever (Smorczewska‐Kiljan et al. 2022; Yoshida et al. 2018). Those children with IVIG resistance were observed to more frequently develop CALs than those without it (35.7% vs. 2.6%) (Kibata et al. 2016). Multivariate logistic regression analysis also identified IVIG resistance as an independent risk factor for the development (Chen et al. 2023) and progression (Xu et al. 2024) of CALs. Major adverse cardiac events (MACE, including myocardial infarction, heart failure, and death) occurred exclusively in patients with larger size of CAA (Friedman et al. 2016; Santimahakullert et al. 2022). Thus, it is very important to stratify KD patients with a higher risk of IVIG resistance and CALs and then early schedule intensified therapy (steroid or cyclosporine) for them to prevent progression and poor prognosis (Lei et al. 2022).

Increasing evidence had shown that most of patients with coronary artery diseases were malnourished and coronary artery disease patients with malnutrition were at a greater risk of all‐cause death (Liu, Huang, Huang, et al. 2022; Wang, Guo, et al. 2021). Albumin (ALB) synthesized by the liver is the main component of the total protein in human serum; hereby, serum ALB concentration has been proposed to represent the nutritional status of populations (Brock et al. 2016). These findings indicate hypoalbuminemia may be a predictor of IVIG resistance and CALs for KD patients. This hypothesis had been demonstrated in the published meta‐analysis studies: Chen et al. (2011) integrated 10 studies to identify that compared with non‐CAL group, serum ALB levels were significantly lower in the CAL group, with the pooled weighted mean difference (WMD) and 95% confidence intervals (CI) of −2.727 (−4.026, −1.427); Baek and Song (2016) (WMD = −0.427; 95% CI: −0.657, −0.198; *p* < 0.001) and Liu, Wang, and Du (2020) (WMD = −0.26; 95% CI: −0.33, −0.20; *p* < 0.001), respectively, pooled 12 and 19 studies to identify that IVIG non‐responders had significantly lower ALB values than responders. However, the associations between ALB and IVIG resistance/CALs of KD patients remained inconclusive because these three studies included the articles published before 2011 (Chen et al. 2011), 2014 (Baek and Song 2016) and 2019 (Liu, Wang, and Du 2020), respectively. Some newly published studies reported the negative associations (Azuma et al. 2020; Duan et al. 2023; Huang et al. 2022; Li, Wu, et al. 2022; Liu, Huang, Chen, et al. 2022). Thus, their conclusions may be overestimated or underestimated. More importantly, all these studies only extracted the mean and standard deviation (SD) to be pooled, which may correspond to the results of univariable analysis, while the odds ratios (ORs) and 95% CIs from multivariable analysis were not collected. Also, the predictive power of ALB based on sensitivity, specificity, diagnostic odds ratio (DOR), and the area under the summary receiver operating characteristic (SROC) curve (AUC) were also not investigated.

Although the etiology of KD is unknown, immune‐mediated proinflammatory mechanisms play important roles (Liu and Wu 2022). Increasing evidence highlights the interconnection between inflammation and nutrition (Mammadov et al. 2020). Therefore, biomarkers that merge the ALB and immune inflammatory parameters, including prognostic nutrition index (PNI) [10 × serum ALB (g/L) + 5 × lymphocyte counts (10^9^/L)] (Li, Wu, et al. 2022; Li et al. 2021; Liu, Su, et al. 2023; Liu, Ye, et al. 2023; Liu, Shao, et al. 2022; Tai et al. 2020; Wang, Ding, et al. 2023), C‐reactive protein/albumin ratio (CAR) (Duan et al. 2023; Li, Wang, Gou, et al. 2020; Liu et al. 2021; Wang, Ding, et al. 2023; Zhang et al. 2023), albumin‐to‐globulin ratio (AGR) (Liu, Su, et al. 2023; Liu, Yue, et al. 2022), and bilirubin‐to‐albumin (BAR) (Liu, Huang, Chen, et al. 2022; Liu, Ye, et al. 2023; Wang, Ding, et al. 2023), may be more accurate in predicting IVIG resistance and CALs for KD patients relative to ALB alone, which was demonstrated previously (AUC of PNI vs. ALB = 0.718 vs. 0.653 for IVIG resistance; Liu, Shao, et al. 2022). Unfortunately, there were also controversies about the associations of these ALB‐combined biomarkers with IVIG resistance and CALs of KD patients in individual studies. Liu, Ye, et al. (2023) found PNI before (*p* = 0.192) and after IVIG (*p* = 0.819) were not significantly independent predictors of IVIG resistance, while Li, Wu, et al. (2022) (*p* = 0.032) and Liu, Shao, et al. (2022) (*p* < 0.001) confirmed PNI as a markedly independent parameter for IVIG resistance. Tai et al. (2020) found the PNI level was significantly lower in the KD patients with CAA present compared with those without CAA, while no significant difference was detected in the study of Liu, Ye, et al. (2023). Tsai et al. (2020) found the CAR was significantly higher in KD children with CALs than those without CALs, while Duan et al. (2023) did not observe significant differences in CAR between CALs and non‐CAL groups. These findings suggested the necessity to perform a meta‐analysis to comprehensively evaluate the associations of ALB‐combined biomarkers with IVIG resistance and CALs of KD patients, which was not reported until now.

Given this background, this present study aimed to perform a meta‐analysis to re‐assess the relationships between all nutrition‐associated biomarkers (pre‐albumin, PA; ALB, PNI, AGR, BAR, and CAR) and the development of IVIG resistance and CALs in KD patients. By integrating all the evidence published before 2023 September, the statistical power was obviously enhanced and thus a more convincing conclusion may be achieved by our study.

## 2 Materials and Methods

### 2.1 Search Strategies

The databases of PubMed, EMBASE, and the Cochrane library were searched to retrieve all relevant articles published up to September 1, 2023, in accordance with the Preferred Reporting Items for Systematic Review and Meta‐analysis (PRISMA) statement. The search keywords and terms included: (“albumin” OR “hypoalbuminemia” OR “hyperalbuminemia” OR “prognostic nutritional index” OR “PNI” OR “C‐reactive protein to albumin ratio” OR “CAR” OR “albumin‐to‐globulin ratio” OR “albumin/globulin ratio” OR “bilirubin‐to‐albumin ratio”) AND (“Kawasaki disease” OR “mucocutaneous lymph node syndrome”) AND (“intravenous immunoglobulin resistance” OR “intravenous immunoglobulin unresponsiveness” OR “coronary artery lesion” OR “cardiovascular complications”). An extensive manual search was also conducted using reference lists of original articles and reviews.

### 2.2 Inclusion and Exclusion Criteria

Studies that satisfied the following inclusion criteria were included: (1) patients were diagnosed as KD; (2) nutrition‐associated biomarkers (PA, ALB, PNI, AGR, BAR, and CAR) were measured; (3) the differences of indicators between KD patients with CALs and non‐CALs, IVIG‐sensitive and IVIG‐resistant were compared; (4) the mean and SD, ORs, and 95% CIs were provided to explore the factors involved in IVIG resistance and CALs, while four‐table data (true positives [TPs], true negatives [TNs], false positives [FPs], and false negatives [FNs]) were described or could be calculated to evaluate the predictive values of identified factors; (5) published in English language; and (6) had full text.

The studies were excluded if they were (1) duplicated articles; (2) case reports, reviews, letters, abstracts, comments, cell or animal studies; (3) data unavailable; and (4) unrelated to the topic of interest.

### 2.3 Data Extraction

Two authors extracted the data independently and disagreements were resolved by discussion. The extracted information included the first author, publication year, country, study design, diagnostic criteria for KD, sample size of cases and controls, indicator acquisition time, laboratory indicators, and their corresponding data (mean and SD, ORs and 95% CIs, TPs, TNs, FPs, and FNs). If ORs and 95% CIs were provided in both univariate and multivariate analyses, the multivariate results (particularly adjusted) were preferentially extracted.

### 2.4 Quality Assessment

The Newcastle‐Ottawa Scale (NOS) (Andreas 2010) that consisted of three domains: patient selection (0–4 points), comparability (0–2 points), and outcome (0–3 points) were applied to assess the methodological quality of all included studies. NOS scores ranged from 0 to 9 points and studies with an NOS score ≥ 7 were regarded as high quality.

### 2.5 Statistical Analysis

The meta‐analysis to verify the associations of potential biomarkers with IVIG resistance and CALs was performed using STATA 15.0 software (STATA Corporation, College Station, TX, USA). The mean and SD of each group in each individual study were pooled to calculate the standardized mean difference (SMD) and 95% CIs. ORs and their 95% CIs from individual studies were pooled to determine the overall risk. The significance of the effect size was estimated by the *Z*‐test, with a *p*‐value < 0.05 as the statistical threshold. The meta‐analysis to confirm the predictive performance of biomarkers was conducted using Meta‐DiSc software (version 1.4; Universidad Complutense, Barcelona, Spain) (Zamora et al. 2006), by which the pooled sensitivity, specificity, DOR, and SROC were obtained. The higher the sensitivity, specificity, and DOR, the better the predictive effects. The AUC of SROC represented a global measurement of test performance. AUC values were graded as the following: 0.9–1.0, excellent; 0.8–0.9, good; 0.7–0.8, fair; 0.6–0.7, poor; 0.5–0.6, bad; < 0.5, useless (Metz 1978). The heterogeneity among the studies was examined using the Cochran's *Q* test and *I* ^2^ statistic. If significant heterogeneity existed among included studies (*p* < 0.1 and *I* ^2^ > 50%), a random‐effect model was chosen to analyze the data; otherwise, a fixed‐effect model was recommended. To explore the potential source of heterogeneity, subgroup analyses were performed for biomarkers with more than five datasets according to country (Asian or non‐Asian), study design (prospective or retrospective; single‐center or multicenter), sample size (< 300 or > 300), indicator acquisition time (pre‐IVIG or post‐IVIG), and source of OR (univariate or multivariate). Furthermore, a multivariable meta‐regression analysis was also conducted for biomarkers with at least 10 datasets to investigate possible heterogenous factors. Egger's linear regression test was applied to investigate the publication bias (Egger et al. 1997). If a publication bias was revealed (*p* < 0.05), the trim‐and‐fill analysis (Peters et al. 2007) was then performed to compute an adjusted effect size. The robustness of meta‐analysis results was confirmed by “leave‐one‐out” sensitivity analyses.

## 3 Results

### 3.1 Study Selection

**FIGURE 1:**
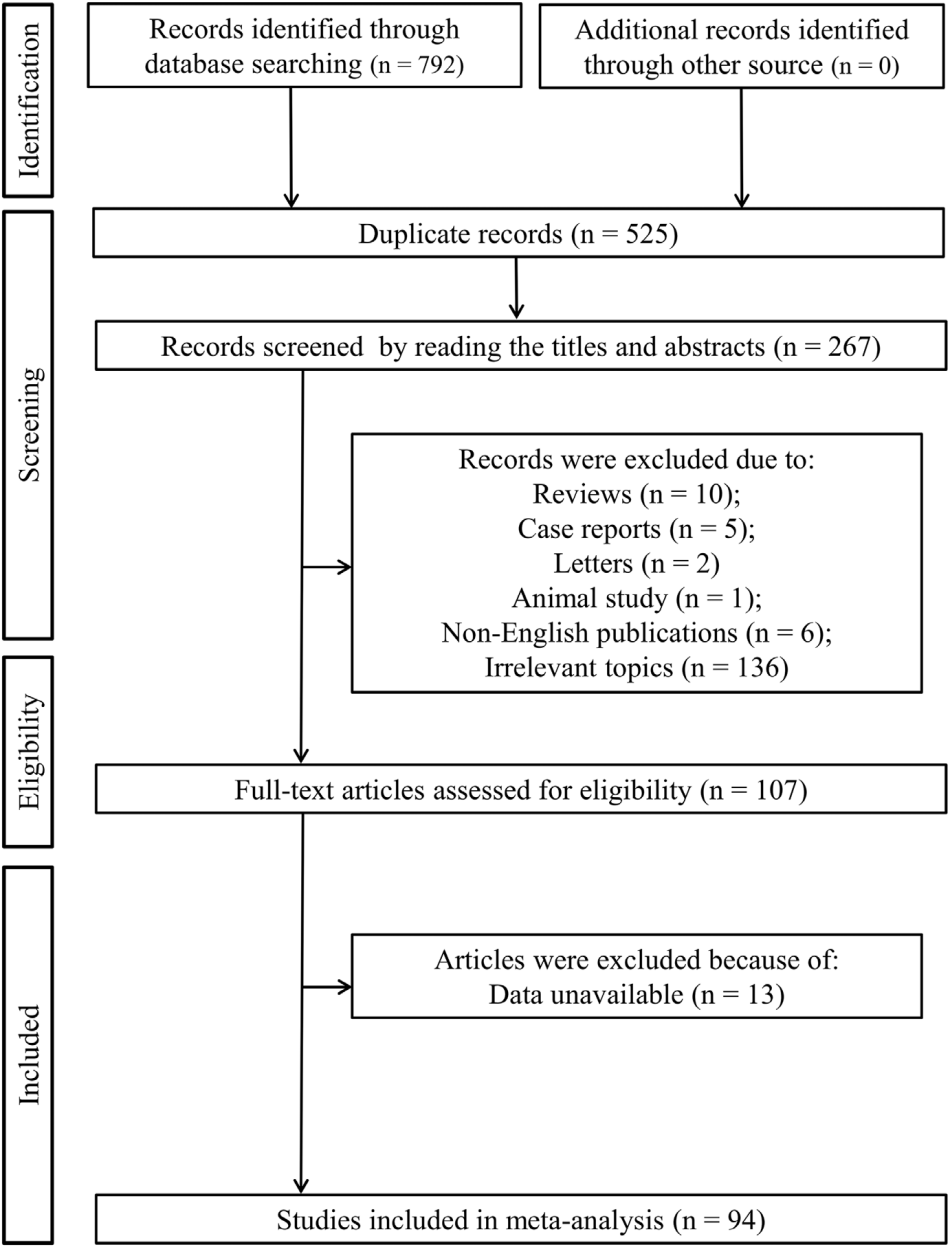
Flowchart of the study selection.

The flow chart diagram of the study selection is displayed in Figure 1. A total of 792 records were initially retrieved by searching the electronic databases; 525 of them were duplicated and thus removed. After reading titles and abstracts, 160 articles were excluded because they were reviews (*n* = 10), case reports (*n* = 5), letters (*n* = 2), animal study (*n* = 1), non‐English publications (*n* = 6), or irrelevant topics (*n* = 136). The full texts of the remaining 106 studies were downloaded and then reviewed. As a result, 13 of them were discarded because of lack of available data. Finally, 94 eligible studies were included for our meta‐analysis (Table 1).

**TABLE 1:** Basic characteristics of included articles.

Author	Year	Country	Design	Diagnostic criteria for KD	Indicator acquisition time	For prediction of IVIG resistance		For prediction of CALs		NOS
						Sample size (IVIG‐resistant vs. IVIG‐sensitive)	Indicators	Sample size (CALs vs. non‐CALs)	Indicators	
Liu, Ye, et al.	2023	China	R, single‐center	AHA	Before and after IVIG	153 (41 vs. 112)	ALB^a^, BAR^a,b,c^, PNI^a,b^, CAR^a,b,c^	153 (37 vs. 116)	ALB^a^, BAR^a^, PNI^a^, CAR^a^	9
Liu, Su, et al.	2023	China	R, single‐center	AHA	Before IVIG			225 (80 vs. 145) (CAA); 261 (116 vs. 145) (CAD); 80 (17 vs. 63) (persistent CAA)	ALB^a,b^, PNI^a,b,c^, CAR^a^, AGR^a,b^	8
Li, Wu, et al.	2022	China	R, single‐center	JKDRC	Before IVIG	635 (47 vs. 588)	ALB^a^, PA^a^, PNI^a,b,c^			9
Yalcinkaya et al.	2022	Turkey	R, single‐center	AHA	Before IVIG			134 (39 vs. 95)	PNI^a,b,c^	8
Liu, Shao, et al.	2022	China	P, single‐center	AHA	Before IVIG	755 (103 vs. 652)	ALB^a,c^, PNI^a,b,c^			8
Li et al.	2021	China	R, multi‐center	AHA	Before IVIG	1257 (187 vs. 1070)	PNI^b,c^			8
Huang et al.	2022	China	R, multi‐center	AHA	Before IVIG	1293 (72 vs. 1221) (training); 205 (14 vs. 191) (test 1)	ALB^a^			9
			P, single‐center	AHA	Before IVIG	209 (6 vs. 203) (test 2)	ALB^a^			9
Mori et al.	2000	Japan	R, single‐center	JKDRC	Before IVIG			193 (24 vs. 169)	ALB^a^	8
Tan et al.	2019	China	R, single‐center	JKDRC	Before IVIG	5277 (348 vs. 4929)	ALB^a,b^, PA^a^			9
Wang, Huang, et al.	2023	China	R, single‐center	JKDRC	Before IVIG	703 (38 vs. 665)	ALB^a,b,c^			9
Wu et al.	2019	China	R, multi‐center	AAP and AHA	Before IVIG	282 (23 vs. 259)	ALB^a,b^			9
Qian et al.	2018	China	P, single‐center	AHA	Before IVIG	504 (25 vs. 479)	ALB^a^			9
Liu, Huang, Chen, et al.	2022	China	R, single‐center	JKDRC	Before IVIG	87 (26 vs. 61)	ALB^a^, BAR^a,b,c^	87 (18 vs. 69) (CAA)	ALB ^a,b,c^, BAR^a^	9
Fu et al.	2013	China	R, single‐center	JKDRC	Before IVIG	1177 (211 vs. 966)	ALB^a,b^			9
Xie, Wang, et al.	2017	China	R, single‐center	JKDRC	Before IVIG	364 (25 vs. 339) (complete); 152 (21 vs. 131) (incomplete)	ALB^a,b,c^	364 (86 vs. 278) (complete); 131 (50 vs. 81) (incomplete)	ALB^a,b,c^	9
Yang et al.	2019	China	R, multi‐center	JKDRC	Before IVIG	1360 (78 vs. 1282)	ALB^a,b^			8
Wang, Ding, et al.	2023	China	R, single‐center	AHA	Before IVIG	1259 (82 vs. 1171) (training); 539 (31 vs. 508) (test1); 177 (21 vs. 156) (test 2)	ALB^a,b^, BAR^a,b^, CAR^a,b^, PNI^a,b^			9
Lu et al.	2021	China	R, single‐center	AHA	Before and after IVIG	94 (40 vs. 54)	ALB^a,b,c^			8
Ahmed et al.	2023	India	P, single‐center	JKDRC	Before IVIG	70 (22 vs. 48)	ALB^a^			7
Hua et al.	2019	China	R, single‐center	JKDRC	Before IVIG			2240 (547 vs. 1693)	ALB^a,b^	9
Wu et al.	2020	China	R, single‐center	AHA	Before IVIG	277 (31 vs. 246)	ALB^a,b^, PA^a^			9
Lao et al.	2022	China	R, single‐center	AHA	Before IVIG			241 (30 vs. 211)	ALB^a,b^	7
Tai et al.	2020	China	R, single‐center	AHA	Before IVIG			275 (149 vs. 126) (CAA)	ALB^a^, PNI^a,b,c^	9
Jing et al.	2022	China	P, single‐center	AHA	Before IVIG	55 (13 vs. 42)	ALB^a^			7
Zhang et al.	2023	China	R, single‐center	AHA	Before IVIG	907 (66 vs. 841)	ALB^a,b,c^, CAR^a,b^			9
Li, Wang, Gou, et al.	2020	China	R, multi‐center	AHA	Before IVIG	957 (159 vs. 798)	ALB^a,b^, CAR^a,b,c^			7
Liu et al.	2021	China	P, single‐center	AHA	Before IVIG	550 (79 vs. 471); 79 (31 vs. 48)	ALB^a,b,c^, CAR^a,b,c^			7
Tsai et al.	2020	China	R, single‐center	AHA	Before IVIG			410 (143 vs. 267)	ALB^a,b^, CAR^a,b,c^	8
Kuo et al.	2010	China	R, single‐center	NA	Before IVIG	95 (15 vs. 80)	ALB^a,b^			7
Yan et al.	2021	China	P, single‐center	AHA	Before IVIG	823 (115 vs. 708)	ALB^a,c^	823 (86 vs. 737)	ALB^a^	8
Song et al.	2009	Korea	R, single‐center	JKDRC	Before IVIG	48 (7 vs. 41)	ALB^a^	161 (21 vs. 141) (< 1 year); 60 (10 vs. 50) (> 5 years)	ALB^a^	9
Lee et al.	2014	Korea	R, single‐center	AHA	Before IVIG	91 (11 vs. 80)	ALB^a^			9
Seo et al.	2016	Korea	R, single‐center	AHA	Before IVIG	80 (9 vs. 71)	ALB^a,b^			9
Yılmazer et al.	2019	Turkey	R, single‐center	AHA	Before IVIG	120 (18 vs. 102)	ALB^a,b^			9
Liu et al.	2017	China	R, single‐center	JKDRC	Before IVIG			169 (31 vs. 138) (CAD); 169 (16 vs. 153) (CAA)	ALB^a,b^	7
Chang et al.	2020	China	R, single‐center	JKDRC	Before IVIG			365 (127 vs. 238)	ALB^a^	7
Sato et al.	2013	Japan	R, single‐center	JKDRC	Before IVIG	105 (21 vs. 84)	ALB^a,b^			7
Fernandez‐Cooke et al.	2019	Spain	R, multi‐center	AHA	Before IVIG	625 (98 vs. 527)	ALB^b^			8
Zhang et al.	2012	China	R, multi‐center	JKDRC, AAP and AHA	Before IVIG			553 (350 vs. 203)	ALB^b^	9
Cho and Kang	2014	Korea	R, single‐center	AHA	Before IVIG	77 (9 vs. 68) (incomplete); 75 (8 vs. 67) (complete)	ALB^a^			9
Sano et al.	2007	Japan	R, multi‐center	NA	Before IVIG	101 (20 vs. 81)	ALB^a^			9
Jarutach et al.	2021	Thailand	R, single‐center	AHA	Before IVIG	130 (17 vs. 113)	ALB^a,b,c^			8
Kuo et al.	2007	China	R, single‐center	AHA	Before IVIG	120 (19 vs. 101)	ALB^a,b^			7
Terai et al.	2003	Japan	R, single‐center	NA	Before and after IVIG	103 (27 vs. 76)	ALB^a^	27 (12 vs. 15)	ALB^a^	9
Lin et al.	2016	China	R, single‐center	AHA	Before IVIG	181 (22 vs. 159)	ALB^a,b^			9
Kobayashi et al.	2006	Japan	R, multi‐center	JKDRC	Before IVIG	546 (112 vs. 434)	ALB^a^			9
Huang et al.	2021	China	R, single‐center	AHA	Before IVIG	84 (8 vs. 76)	ALB^a^			8
Zhao et al.	2016	China	R, single‐center	AHA	Before IVIG			2331 (625 vs. 1706) (CAD); 2331 (215 vs. 2116) (CAA)	ALB^b^	9
Durongpisitkul et al.	2003	Thailand	R, single‐center	AHA	Before IVIG	120 (14 vs. 106)	ALB^a^			9
Masuzawa et al.	2015	Japan	R, multi‐center	JKDRC	Before and after IVIG			44 (15 vs. 29)	ALB^a,b^	8
Yang, Wu, et al.	2023	China	R, multi‐center	AHA	Before IVIG			388 (200 vs. 188)	ALB^b^	8
Bar‐Meir et al.	2018	Israel	R, multi‐center	AHA	Before IVIG	312 (42 vs. 270)	ALB^a^			9
Nakagama et al.	2016	Japan	R, multi‐center	JKDRC	Before and after IVIG	171 (54 vs. 117)	ALB^a^			9
Moon et al.	2016	Korea	R, single‐center	JKDRC	Before IVIG	91 (23 vs. 68)	ALB^a^			9
Park et al.	2013	Korea	R, multi‐center	AHA	Before IVIG	309 (30 vs. 279)	ALB^a^			7
Kim et al.	2018	Korea	R, multi‐center	AHA	Before IVIG	5151 (524 vs. 4627)	ALB^a,b,c^	5151 (524 vs. 4627)	ALB^a,b,c^	9
Kim et al.	2016	Korea	R, single‐center	NA	Before IVIG	703 (118 vs. 585)	ALB^a,b^	703 (240 vs. 463) (CAD)	ALB^a^	9
Kim et al.	2011	Korea	P, single‐center	AHA	Before and after IVIG	129 (22 vs. 107)	ALB^a^			9
Egami et al.	2006	Japan	R, single‐center	JKDRC	Before IVIG	320 (41 vs. 279)	ALB^a^			9
Duan et al.	2023	Korea	P, single‐center	AHA	Before IVIG	93 (11 vs. 82)	ALB^a^, CAR^a^	93 (19 vs. 74)	ALB^a^, CAR^a^	9
Kitoh et al.	2021	Japan	P, single‐center	JKDRC	Before IVIG	68 (22 vs. 46)	ALB^a^	75 (24 vs. 51)	ALB^a^	9
Wang et al.	2016	China	P, single‐center	AHA	Before IVIG	686 (111 vs. 575)	ALB^a^			7
Chantasiriwan et al.	2018	Thailand	R, single‐center	AHA	Before IVIG	217 (26 vs. 191)	ALB^a^	217 (55 vs. 162) (CAA)	ALB^a^	9
Kaneko et al.	2011	Japan	P, single‐center	JKDRC	Before IVIG			43 (6 vs. 37)	ALB^a^	9
Yoshimura et al.	2013	Japan	P, single‐center	JKDRC	Before IVIG	80 (17 vs. 63)	ALB^a^	80 (19 vs. 61)	ALB^a^	9
Xue and Wang	2020	China	P, single‐center	AHA	Before IVIG			102 (47 vs. 55)	ALB^a^	9
Peng et al.	2019	China	P, single‐center	JKDRC	Before IVIG			58 (30 vs. 28)	ALB^a^	9
Sánchez‐Manubens et al.	2016	Spain	R, multi‐center	AHA	Before IVIG	399 (67 vs. 332)	ALB^a^			9
Tang, Yan, et al.	2016	China	R, single‐center	AHA	Before IVIG	910 (46 vs. 864)	ALB^a,b^			7
Gámez‐González et al.	2018	México	R, single‐center	NA	Before IVIG	419 (101 vs. 318)	ALB^a,b^			9
Arane et al.	2018	Israel	R, multi‐center	NA	Before IVIG	282 (52 vs. 230)	ALB^a,b^			7
Fabi et al.	2018	Italy	R, multi‐center	AHA	Before IVIG			302 (14 vs. 288)	ALB^b^	7
Sleeper et al.	2011	Canada	P, multi‐center	AHA	Before IVIG		ALB^a^	182 (27 vs. 155)	ALB^a,b^	8
Davies et al.	2015	UK	R, single‐center	JKDRC	Before IVIG	59 (19 vs. 40)	ALB^a^	59 (20 vs. 39)	ALB^a^	8
Koliou et al.	2023	Cyprus	R, multi‐center	AHA	Before IVIG		ALB^a^	129 (21 vs. 108)	ALB^b^	9
Li, Xu, et al.	2022	China	R, single‐center	AHA	Before IVIG	261 (51 vs. 210)	ALB^a,b^	261 (42 vs. 219)	ALB^a^	9
Do et al.	2010	Korea	R, single‐center	JKDRC	Before IVIG	77 (13 vs. 64)	ALB^a^			8
Xie et al.	2020	China	R, multi‐center	JKDRC	Before IVIG			4276 (374 vs. 3902)	ALB^a,b^	8
Cheng et al.	2022	China	R, single‐center	JKDRC	Before IVIG			71 (33 vs. 38) (CAA)	ALB^a^	9
Singh et al.	2022	USA	R, single‐center	AHA	Before IVIG	208 (39 vs. 169)	ALB^a^			7
Jiang et al.	2024	China	R, single‐center	JKDRC	Before IVIG			1331 (63 vs. 1268) (training); 193 (17 vs. 176) (test)	ALB^a,b^	8
Ohno et al.	2002	Japan	R, single‐center	NA	Before IVIG			40 (5 vs. 35)	ALB^a^	9
Luo et al.	2019	China	P, single‐center	AHA	Before IVIG			118 (56 vs. 62)	ALB^a^	9
Li, Wang, Li, et al.	2020	China	P, single‐center	AHA	Before IVIG	131 (30 vs. 101)	ALB^a,b^, PA^a,b^	131 (18 vs. 113)	ALB^a,b^	7
Wang, Yang, et al.	2021	China	R, single‐center	JKDRC	Before IVIG			130 (76 vs. 54)	ALB^a,b^	8
Honkanen et al.	2003	Canada	P, single‐center	NA	Before IVIG			342 (98 vs. 246) (CAL); 344 (23 vs. 321) (CAA)	ALB^a^	8
Kim et al.	2012	Korea	P, multi‐center	AHA	Before IVIG	478 (47 vs. 431)	ALB^a,b^	478 (159 vs. 319)	ALB^a,b^	8
Yang, Mao, et al.	2023	China	R, single‐center	AHA	Before IVIG	1490 (127 vs. 1363)	ALB^a,b^	1490 (143 vs. 1347)	ALB^a,b^	7
Liu, Yue, et al.	2022	China	R, single‐center	JKDRC	Before IVIG			446 (92 vs. 354) (small‐sized CAA); 392 (38 vs. 354) (mid‐ to large‐sized CAA)	ALB^a,b^, AGR^a,b^, CAR^a^	8
Tang, Gao, et al.	2016	China	R, single‐center	AHA	Before IVIG			1004 (240 vs. 764)	ALB^a,b^	7
Azuma et al.	2020	Japan	R, single‐center	JKDRC	Before IVIG	72 (11 vs. 61) (initial); 27 (11 vs. 15) (second)	ALB^a^			9
Hester et al.	2019	USA	R, single‐center	AHA	Before IVIG	430 (81 vs. 349)	ALB^a,b^			9
Seo et al.	2018	Korea	R, multi‐center	JKDRC, AHA	Before IVIG			615 (119 vs. 496)	ALB^a^	7
Mammadov et al.	2020	China	R, single‐center	AHA	Before IVIG			210 (19 vs. 191)	ALB^a,b^, AGR^a,b^	8

*Note:* Data were expressed as mean (standard deviation)^a^ or odds ratios (95% confidence intervals)^b^ for association analyses and four‐table data (true positives; false positives; false negatives; true negatives)^c^ for predictive analyses. CALs included CAA and CAD.

Abbreviations: AAP, American Academy of Pediatrics; AGR, albumin‐to‐globulin ratio; AHA, American Heart Association; ALB, albumin; BAR, total bilirubin‐to‐albumin ratio; CAA, coronary artery aneurysm; CAD, coronary artery dilatation; CALs, coronary artery lesions; CAR, C‐reactive protein to albumin ratio; IVIG, intravenous immunoglobulin; JKDRC, Japanese Kawasaki Disease Research Committee; KD, Kawasaki disease; NA, not available; P, prospective; PA, pre‐albumin; PNI, prognostic nutritional index; R, retrospective.

### 3.2 Study Characteristics

The characteristics of included studies are shown in Table 1. These 94 studies were officially published from 2000 to 2024. The patients in them were enrolled from 14 countries, including China (*n* = 50), Japan (*n* = 13), Korea (*n* = 13), Thailand (*n* = 3), Canada (*n* = 2), Turkey (*n* = 2), Spain (*n* = 2), USA (*n* = 2), Israel (*n* = 2), México (*n* = 1), Italy (*n* = 1), India (*n* = 1), Cyprus (*n* = 1), and UK (*n* = 1). Seventy‐five studies were retrospective and 19 studies were prospective, 22 studies were designed as a multicenter trial, and 71 were a single‐center trial; one study (Huang et al. 2022) included a retrospective multicenter trial (training and test 1 datasets) and a prospective single‐center trial (test 2). KD was diagnosed according to the guidelines of the American Heart Association (AHA), American Academy of Pediatrics (AAP), and/or Japanese Kawasaki Disease Research Committee (JKDRC). Six did not definitely describe that diagnostic criteria was used for KD. Most of studies collected the laboratory data before IVIG treatment and only six studies obtained the data before and after IVIG treatment. Forty‐eight studies explored the biomarkers associated with IVIG resistance, 30 investigated the factors contributing to the development of CALs and 17 evaluated laboratory predictors for both IVIG resistance and CALs. The NOS scores in all studies were ≥ 7, indicating they were of high methodological quality.

### 3.3 Meta‐Analysis to Screen Predictors for IVIG Resistance of KD Patients

#### 3.3.1 ABL

**FIGURE 2:**
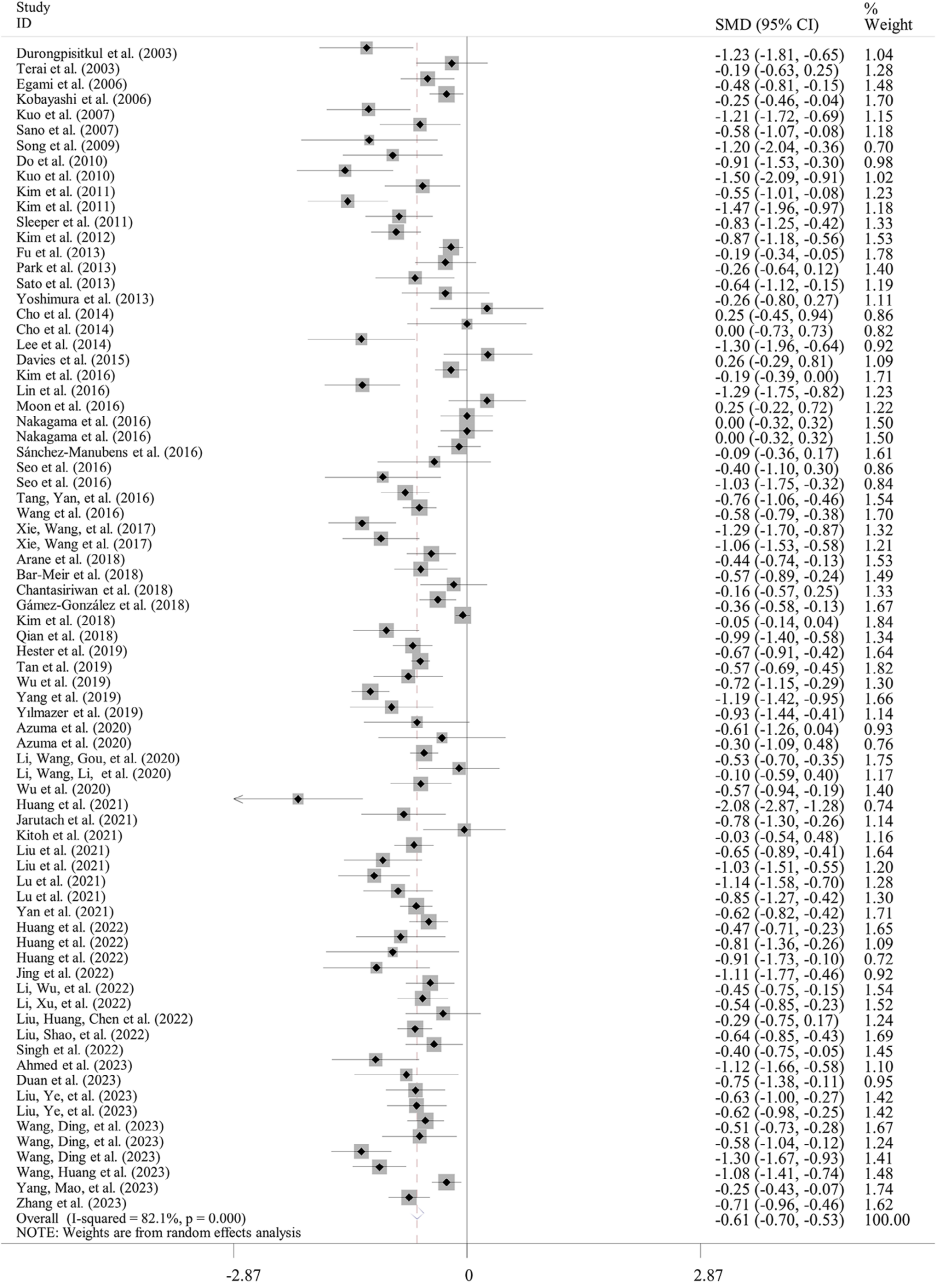
Meta‐analysis of differences in albumin level between IVIG‐resistant and IVIG‐sensitive KD patients. CIs, confidence intervals; IVIG, intravenous immunoglobulin; KD, Kawasaki disease; SMD, standardized mean difference.

Sixty‐three studies with 76 datasets compared the ABL levels between IVIG‐resistant and IVIG‐sensitive KD patients. Random‐effect model was applied for the meta‐analysis since significant heterogeneity was detected (*I* ^2^ = 82.1%; *p* < 0.001). The pooled analysis showed that compared with the IVIG‐sensitive group, the ABL level was significantly lower in the IVIG‐resistant ones (SMD = −0.61; 95% CI: −0.70, −0.53; *p* < 0.001) (Table 2; Figure 2). The result was still significant in subgroup analyses based on country (Asian: SMD = −0.64, *p* < 0.001; non‐Asian: SMD = −0.43, *p* = 0.001), design (prospective: SMD = −0.71; retrospective: SMD = −0.59; multicenter: SMD = −0.47; single‐center: SMD = −0.66; *p* < 0.001), sample size (< 300: SMD = −0.65; > 300: SMD = −0.58; *p* < 0.001), and indicator acquisition time (pre‐IVIG: SMD = −0.61, *p* < 0.001; post‐IVIG: SMD = −0.71, *p* = 0.017) (Table 3). Unfortunately, the source of heterogeneity could not be confirmed according to these subgroup factors because the random‐effect model was still used for analysis of all of them. Meta‐regression analysis was further conducted; however, no factors were also found to account for the heterogeneity (Table 4).

**TABLE 2:** Meta‐analysis to evaluate the associations of biomarkers with IVIG resistance and CALs of KD patients.

Variables			No.	ES	95% CI	*P*_E_	*I* ^2^	*P*_H_	Model	Egger *p*
ALB	IVIG resistance	SMD	76	−0.61	−0.70, −0.53	< 0.001	82.1	< 0.001	R	< 0.001
		OR	40	0.83	0.79, 0.88	< 0.001	84.8	< 0.001	R	< 0.001
	CALs	SMD	51	−0.56	−0.74, −0.39	< 0.001	95.4	< 0.001	R	0.075
		OR	26	0.92	0.87, 0.96	0.001	84.9	< 0.001	R	0.001
PA	IVIG resistance	SMD	4	−0.72	−1.17, −0.26	0.002	89.3	< 0.001	R	0.052
PNI	IVIG resistance	SMD	7	−0.34	−0.96, 0.29	0.290	96.6	< 0.001	R	0.212
		OR	6	1.04	0.99, 1.09	0.149	91.5	< 0.001	R	0.239
	CALs	SMD	7	−0.20	−0.40, 0.01	0.052	61.9	0.015	R	0.152
		OR	4	0.82	0.72, 0.94	0.003	71.6	0.014	R	0.379
BAR	IVIG resistance	SMD	6	1.10	0.49, 1.72	< 0.001	94.1	< 0.001	R	0.518
		OR	3	1.99	0.66, 6.02	0.226	77.3	0.012	R	0.024
	CALs	SMD	3	0.13	−0.11, 0.36	0.285	40.4	0.187	F	0.584
CAR	IVIG resistance	SMD	10	1.47	0.95, 2.00	< 0.001	96.2	< 0.001	R	0.500
		OR	7	1.69	1.39, 2.05	< 0.001	72.6	0.001	R	0.854
	CALs	SMD	9	0.52	0.21, 0.84	0.001	89.4	< 0.001	R	0.047
AGR	CALs	SMD	6	−0.19	−0.39, 0.01	0.066	56.7	0.042	R	0.185
		OR	3	0.82	0.34, 1.96	0.655	0.0	0.883	F	0.157

Abbreviations: AGR, albumin‐to‐globulin ratio; ALB, albumin; BAR, total bilirubin‐to‐albumin ratio; CALs, coronary artery lesions; CAR, C‐reactive protein to albumin ratio; CI, confidence interval; ES, effect size; F, fixed‐effect; IVIG, intravenous immunoglobulin; OR, odds ratio; PA, pre‐albumin; *P*_E_, significance for effects; *P*_H_, significance for heterogeneity; PNI, prognostic nutritional index; R, random‐effect; SMD, standardized mean difference.

**TABLE 3:** Subgroup analysis to evaluate the associations of biomarkers with IVIG resistance and CALs of KD patients.

Variables					No.	ES	95% CI	*P*_E_	*I* ^2^	*P*_H_	Model
ALB	IVIG resistance	SMD	Country	Asian	69	−0.64	−0.73, −0.54	< 0.001	82.8	< 0.001	R
				Non‐Asian	7	−0.43	−0.68, −0.18	0.001	74.5	0.001	R
			Design	Prospective	15	−0.71	−0.86, −0.56	< 0.001	61.0	0.001	R
				Retrospective	61	−0.59	−0.69, −0.49	< 0.001	83.2	< 0.001	R
				Multicenter	15	−0.47	−0.66, −0.27	< 0.001	88.8	< 0.001	R
				Single‐center	60	−0.66	−0.75, −0.56	< 0.001	75.1	< 0.001	R
			Sample size	< 300	48	−0.65	−0.78, −0.52	< 0.001	71.6	< 0.001	R
				> 300	28	−0.58	−0.69, −0.45	< 0.001	88.7	< 0.001	R
			Indicator acquisition time	Pre‐IVIG	72	−0.61	−0.70, −0.52	< 0.001	81.9	< 0.001	R
				Post‐IVIG	4	−0.71	−1.30, −0.13	0.017	88.6	< 0.001	R
		OR	Country	Asian	33	0.87	0.83, 0.92	< 0.001	82.9	< 0.001	R
				Non‐Asian	7	0.31	0.17, 0.57	< 0.001	84.2	< 0.001	R
			Design	Prospective	6	0.43	0.23, 0.80	0.008	83.5	< 0.001	R
				Retrospective	34	0.84	0.80, 0.89	< 0.001	85.4	< 0.001	R
				Multi‐center	10	0.53	0.41, 0.68	< 0.001	85.2	< 0.001	R
				Single‐center	30	0.88	0.83, 0.92	< 0.001	84.0	< 0.001	R
			Sample size	< 300	21	0.56	0.46, 0.67	< 0.001	81.6	< 0.001	R
				> 300	19	0.89	0.84, 0.94	< 0.001	86.8	< 0.001	R
			Indicator acquisition time	Pre‐IVIG	39	0.83	0.79, 0.88	< 0.001	85.1	< 0.001	R
				Post‐IVIG	1	0.45	0.15, 1.32	0.144	—	—	R
			OR source	UV	6	0.93	0.80, 1.07	0.299	75.0	0.001	R
				MV	34	0.80	0.75, 0.85	< 0.001	85.8	< 0.001	R
	CALs	SMD	Country	Asian	48	−0.59	−0.78, −0.41	< 0.001	95.6	< 0.001	R
				Non‐Asian	3	−0.06	−0.87, 0.75	0.877	92.0	0.001	R
			Design	Prospective	11	−0.79	−1.18, −0.39	< 0.001	92.6	0.001	R
				Retrospective	40	−0.50	−0.70, −0.31	< 0.001	95.7	< 0.001	R
				Multi‐center	6	−0.03	−0.37, 0.32	0.883	95.1	< 0.001	R
				Single‐center	45	−0.65	−0.86, −0.45	< 0.001	95.1	< 0.001	R
			Sample size	< 300	34	−0.45	−0.68, −0.23	< 0.001	88.7	< 0.001	R
				> 300	17	−0.75	−1.05, −0.46	< 0.001	98.0	< 0.001	R
			Indicator acquisition time	Pre‐IVIG	48	−0.64	−0.81, −0.46	< 0.001	95.4	< 0.001	R
				Post‐IVIG	3	1.22	−0.94, 3.37	0.270	96.4	< 0.001	R
			CAL type	CAA	16	−0.62	−0.91, −0.33	< 0.001	9.5	< 0.001	R
				CAD	3	−0.45	−1.07, 0.18	0.164	94.6	< 0.001	R
				Total CALs	32	−0.54	−0.78, −0.31	< 0.001	96.5	< 0.001	R
		OR	Country	Asian	23	0.93	0.89, 0.98	0.004	84.6	< 0.001	R
				Non‐Asian	3	0.33	0.21, 0.53	< 0.001	0.0	0.399	F
			Design	Prospective	4	0.57	0.30, 1.06	0.076	78.4	0.003	R
				Retrospective	22	0.93	0.88, 0.97	0.003	78.4	0.003	R
				Multi‐center	7	0.91	0.83, 1.00	0.050	88.1	< 0.001	R
				Single‐center	19	0.90	0.83, 0.97	0.006	83.4	< 0.001	R



			Sample size	< 300	10	0.64	0.46, 0.88	0.006	76.8	< 0.001	R
				> 300	16	0.94	0.89, 0.98	0.008	87.5	< 0.001	R
			OR source	UV	7	0.63	0.42, 0.94	0.022	80.5	< 0.001	R
				MV	19	0.92	0.88, 0.97	0.002	86.7	< 0.001	R
CAL type			CAA	7	0.60	0.39, 0.92	0.018	85.1	< 0.001	R
	CAD		1	1.14	0.97, 1.35	0.106	—	—	R
	Total CALs	18	0.93	0.89, 0.98	0.005	84.5	< 0.001	R
PNI	IVIG resistance	SMD	Design	Prospective	1	−1.26	−1.47, −1.04	< 0.001	—	—	R
				Retrospective	6	−0.18	−0.86, 0.50	0.605	96.0	< 0.001	R
			Sample size	< 300	3	0.34	−1.09, 0.78	0.638	97.4	< 0.001	R
				> 300	4	−0.82	−1.42, −0.23	0.007	94.9	< 0.001	R
			Indicator acquisition time	Pre‐IVIG	6	−0.32	−1.04, 0.40	0.385	97.1	< 0.001	R
				Post‐IVIG	1	−0.42	−0.79, −0.06	0.021	—	—	R
		OR	Design	Prospective	1	1.07	1.03, 1.10	< 0.001	—	—	R
				Retrospective	5	1.03	0.97, 1.10	0.306	91.4	< 0.001	R
				Multi‐center	1	1.17	1.12, 1.023	< 0.001	—	—	R
				Single‐center	5	1.01	0.97, 1.05	0.641	83.2	< 0.001	R
			Sample size	< 300	2	0.99	0.94, 1.03	0.516	58.6	0.120	R
				> 300	4	1.08	0.99, 1.18	0.073	94.3	< 0.001	R
			OR source	UV	1	1.00	0.98, 1.01	0.500	—	—	R
				MV	5	1.05	0.98, 1.13	0.156	91.1	< 0.001	R
			Indicator acquisition time	Pre‐IVIG	5	1.05	0.98, 1.13	0.167	93.1	< 0.001	R
				Post‐IVIG	1	1.00	0.97, 1.03	0.793	—	—	R
	CALs	SMD	Country	Asian	6	−0.13	−0.32, 0.05	0.162	—	—	R
				Non‐Asian	1	−0.57	−0.95, −0.19	0.003	52.9	0.060	R
			Indicator acquisition time	Pre‐IVIG	6	−0.23	−0.46, −0.00	0.050	67.2	0.009	R
				Post‐IVIG	1	−0.03	−0.40, 0.34	0.876	—	—	R
			CAL type	CAA	5	−0.18	−0.33, −0.04	0.013	41.6	0.144	F
				CAD	1	0.10	−0.15, 0.34	0.432	—	—	F
				Total CALs	1	−0.57	−0.95, −0.19	0.003	—	—	F
BAR	IVIG resistance	SMD	Sample size	< 300	4	0.84	0.55, 1.13	< 0.001	47.8	0.125	F
				> 300	2	1.65	−0.33, 3.63	0.103	98.7	< 0.001	R
			Indicator acquisition time	Pre‐IVIG	5	1.14	0.38, 1.89	0.003	95.3	0.000	R
				Post‐IVIG	1	0.93	0.56, 1.30	< 0.001	—	—	R
CAR	IVIG resistance	SMD	Design	Prospective	2	0.80	0.58, 1.01	< 0.001	0.0	0.456	F
				Retrospective	8	1.63	1.06, 2.21	< 0.001	95.9	< 0.001	R
				Multi‐center	1	2.56	2.35, 2.76	< 0.001	—	—	R
				Single‐center	9	1.35	0.88, 1.81	< 0.001	93.7	< 0.001	R
			Sample size	< 300	5	1.37	0.41, 2.33	0.005	95.0	< 0.001	R
				> 300	5	1.57	0.88, 2.26	< 0.001	97.3	< 0.001	R
			Indicator acquisition time	Pre‐IVIG	9	1.31	0.78, 1.83	0.003	96.1	< 0.001	R
				Post‐IVIG	1	3.03	2.53, 3.52	< 0.001	—	—	R
		OR	Design	Prospective	2	2.56	1.40, 4.70	0.002	9.8	0.292	F
				Retrospective	5	1.62	1.33, 1.98	< 0.001	79.0	0.001	R
				Multi‐center	1	1.33	1.11, 1.60	0.002	—	—	R
				Single‐center	6	1.81	1.51, 2.17	< 0.001	57.9	0.037	R
			Sample size	< 300	2	2.14	0.74, 6.18	0.161	71.1	0.063	R
				> 300	5	1.72	1.39, 2.13	< 0.001	75.0	0.003	R
			OR source	UV	1	2.05	1.78, 2.37	< 0.001	—	—	R
				MV	6	1.59	1.28, 1.98	< 0.001	65.1	0.013	R
			Indicator acquisition time	Pre‐IVIG	6	1.77	1.42, 2.20	< 0.001	72.6	0.003	R
				Post‐IVIG	1	1.42	1.13, 1.77	0.002	—	—	R
	CALs	SMD	Sample size	< 300	6	0.56	0.13, 0.99	0.011	91.3	< 0.001	R
				> 300	3	0.47	−0.09, 1.03	0.103	87.2	< 0.001	R
			Indicator acquisition time	Pre‐IVIG	8	0.41	0.13, 0.70	0.005	85.6		R
				Post‐IVIG	1	1.36	0.96, 1.76	< 0.001	—	—	R
			CAL type	CAA	6	0.59	0.17, 1.00	0.006	89.1	< 0.001	R
				CAD	1	−0.19	−0.43, 0.06	0.129	—	—	R
				Total CALs	2	0.75	0.01, 1.48	0.047	85.3	0.009	R
AGR	CALs	SMD	Sample size	< 300	4	−0.12	−0.29, 0.06	0.189	8.9	0.349	F
				> 300	2	−0.28	−0.85, 0.29	0.340	87.4	0.005	R
			CAL type	CAA	4	−0.27	−0.57, 0.02	0.068	70.2	0.018	R
				CAD	1	−0.02	−0.27, 0.22	0.869	—	—	R
				Total CALs	1	−0.08	−0.55, 0.39	0.745	—	—	R

Abbreviations: AGR, albumin‐to‐globulin ratio; ALB, albumin; BAR, total bilirubin‐to‐albumin ratio; CAA, coronary artery aneurysm; CAD, coronary artery dilatation; CALs, coronary artery lesions; CAR, C‐reactive protein to albumin ratio; CI, confidence interval; F, fixed effect; IVIG, intravenous immunoglobulin; MV, multivariable analysis; OR, odds ratio; *P*_E_, significance for effects; *P*_H_, significance for heterogeneity; PNI, prognostic nutritional index; R, random‐effect; SMD, standardized mean difference; UV, univariable analysis.

**TABLE 4:** Meta‐regression analysis.

Moderator variables					Coef.	SE	*Z*	*p*	95% CI
ALB	IVIG resistance	SMD	Country	Asian	0.059	0.091	0.65	0.516	−0.119, 0.237
				Non‐Asian					
			Design	Prospective	−0.008	0.077	−0.10	0.920	−0.158, 0.143
				Retrospective					
				Multi‐center	0.041	0.097	0.42	0.672	−0.150, 0.232
				Single‐center					
			Sample size	< 300	−1.151	0.106	−1.42	0.154	−0.359, 0.057
				> 300					
			Indicator acquisition time	Pre‐IVIG	−0.049	0.476	−0.10	0.917	−0.982, 0.883
				Post‐IVIG					
		OR	Country	Asian	0.094	0.225	0.42	0.675	−0.346, 0.535
				Non‐Asian					
			Design	Prospective	−0.014	0.053	−0.26	0.791	−0.117, 0.089
				Retrospective					
				Multi‐center	−0.033	0.073	−0.45	0.653	−0.176, 0.110
				Single‐center					
			Sample size	< 300	0.009	0.042	−0.21	0.837	−0.091, 0.074
				> 300					
			Indicator acquisition time	Pre‐IVIG	0.519	0.801	0.65	0.517	−1.052, 2.090
				Post‐IVIG					
			OR source	UV	0.007	0.039	0.17	0.868	−0.070, 0.083
				MV					
	CALs	SMD	Country	Asian	0.010	0.330	0.03	0.975	−0.636, 0.657
				Non‐Asian					
			Design	Prospective	−0.051	0.144	−0.36	0.721	−0.333, 0.231
				Retrospective					
				Multi‐center	0.036	0.079	0.46	0.649	−0.119, 0.191
				Single‐center					
			Sample size	< 300	−0.118	0.077	−1.53	0.125	−0.268, 0.033
				> 300					
			Indicator acquisition time	Pre‐IVIG	−0.016	0.065	−0.25	0.801	−0.144, 0.112
				Post‐IVIG					
			CAL type	CAA	−0.003	0.052	−0.05	0.958	−0.105, 0.099
				CAD					
				Total CALs					
		OR	Country	Asian	0.194	0.635	0.31	0.760	−1.050, 1.438
				Non‐Asian					
			Design	Prospective	0.644	0.130	4.94	< 0.001	0.389, 0.900
				Retrospective					
				Multi‐center	0.020	0.013	1.55	0.121	−0.005, 0.046
				Single‐center					



			Sample size	< 300	−0.614	0.080	−7.70	< 0.001	−0.771, −0.456
				> 300					
			OR source	UV	−0.130	0.108	−1.21	0.227	−0.342, 0.081
				MV					
CAL type			CAA	−0.113	0.053	−2.12	0.034	−0.216, −0.009
	CAD							
	Total CALs							
CAR	IVIG resistance	SMD	Design	Prospective	0.003	0.723	0.00	0.997	−1.414, 1.420
				Retrospective					
				Multicenter	0.030	2.645	0.01	0.991	−5.155, 5.215
				Single‐center					
			Sample size	< 300	−0.098	0.673	−0.15	0.884	−1.418, 1.222
				> 300					
			Indicator acquisition time	Pre‐IVIG	0.020	3.029	0.01	0.995	−5.916, 5.956
				Post‐IVIG					
		OR	Design	Prospective	0.209	0.799	0.26	0.794	−1.357, 1.775
				Retrospective					
				Multi‐center	0.436	0.373	1.17	0.243	−0.296, 1.168
				Single‐center					
			Sample size	< 300	−0.273	1.658	−0.16	0.869	−3.521, 2.976
				> 300					
			OR source	UV	0.456	0.759	0.60	0.548	−1.032, 1.944
				MV					
			Indicator acquisition time	Pre‐IVIG	−0.688	1.711	−0.40	0.688	−4.041, 2.665
				Post‐IVIG					
	CALs	SMD	Sample size	< 300	−0.032	0.187	−0.17	0.865	−0.398, 0.335
				> 300					
			Indicator acquisition time	Pre‐IVIG	0.055	1.37	0.04	0.968	−2.632, 2.743
				Post‐IVIG					
			CAL type	CAA	−0.018	0.191	−0.09	0.926	−0.393, 0.357
				CAD					
				Total CALs					

Abbreviations: ALB, albumin; CAA, coronary artery aneurysm; CAD, coronary artery dilatation; CALs, coronary artery lesions; CAR, C‐reactive protein to albumin ratio; CI, confidence interval; IVIG, intravenous immunoglobulin; MV, multivariable analysis; OR, odds ratio; SMD, standardized mean difference; Std, standard; UV, univariable analysis.

**FIGURE 3:**
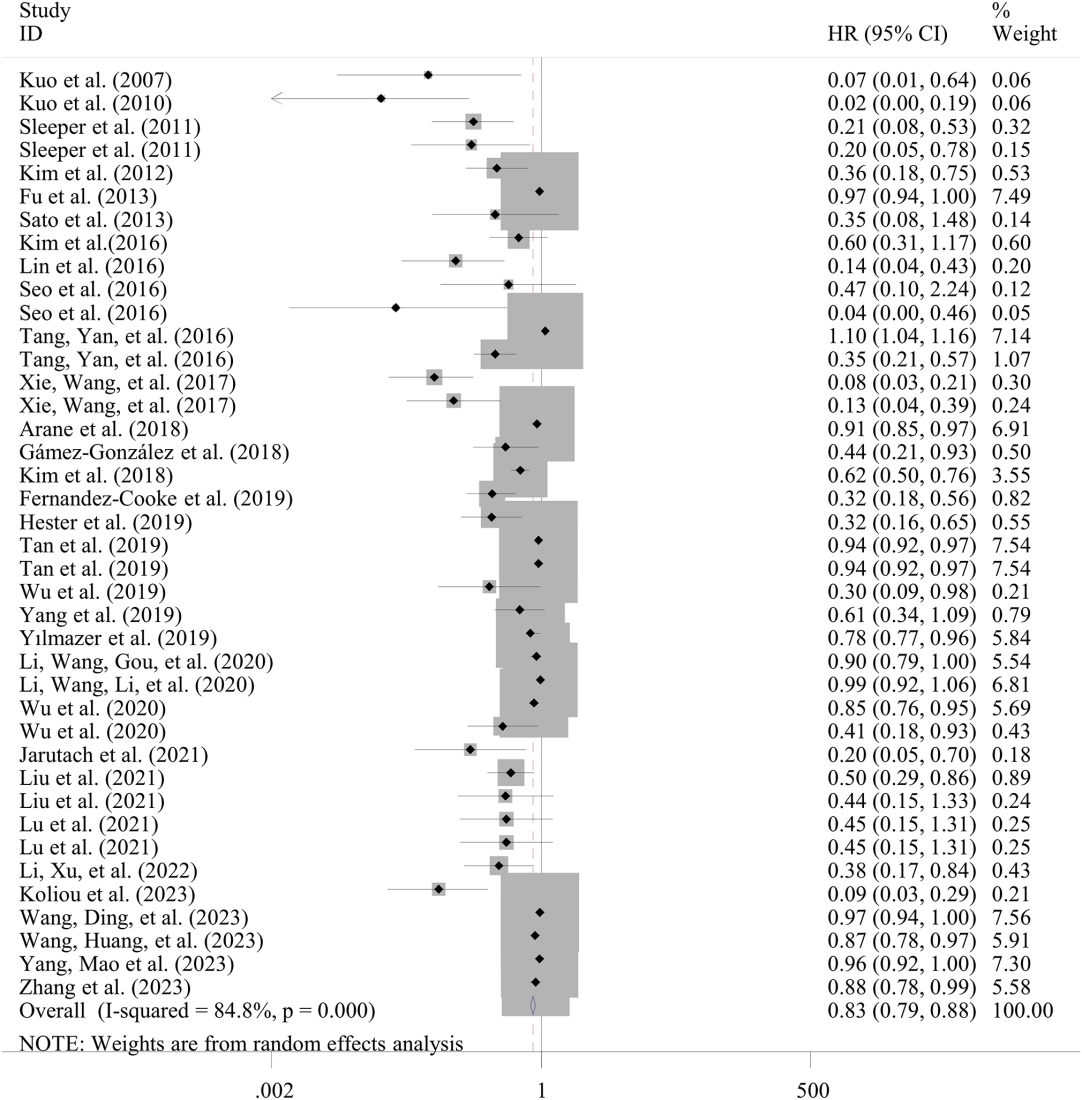
Meta‐analysis of the risk to develop IVIG resistance in KD patients when comparing the high albumin level group with the low albumin level group. CIs, confidence intervals; IVIG, intravenous immunoglobulin; KD, Kawasaki disease; OR, odds ratio.

Thirty‐two studies with 40 datasets reported the association between ABL levels and the risk of IVIG resistance in KD patients. Under the random‐effect model (*I* ^2^ = 84.8%; *p* < 0.001), the meta‐analysis showed that a high ABL level was associated with a decreased risk of IVIG resistance (OR = 0.83, 95% CI: 0.79–0.88, *p* < 0.001) (Table 2; Figure 3). Subgroup analysis demonstrated pre‐IVIG levels of ABL may be an independent predictor for IVIG resistance by pooling of multivariable data (OR = 0.80, *p* < 0.001), but not univariable results (*p* = 0.299) (Table 3). Subgroup factors of country (Asian: OR = 0.87; non‐Asian: OR = 0.31; *p* < 0.001), design (prospective: OR = 0.43, *p* = 0.008; retrospective: OR = 0.84; multicenter: OR = 0.53; single‐center: OR = 0.88; other *p* < 0.001), and sample size (< 300: OR = 0.56; > 300: OR = 0.89; *p* < 0.001) did not change the significant results (Table 3), but the causes of heterogeneity also remained unclear because the random‐effect model was used for analysis of them, and *p*‐value was > 0.05 for all variables in meta‐regression analysis (Table 4).

**FIGURE 4:**
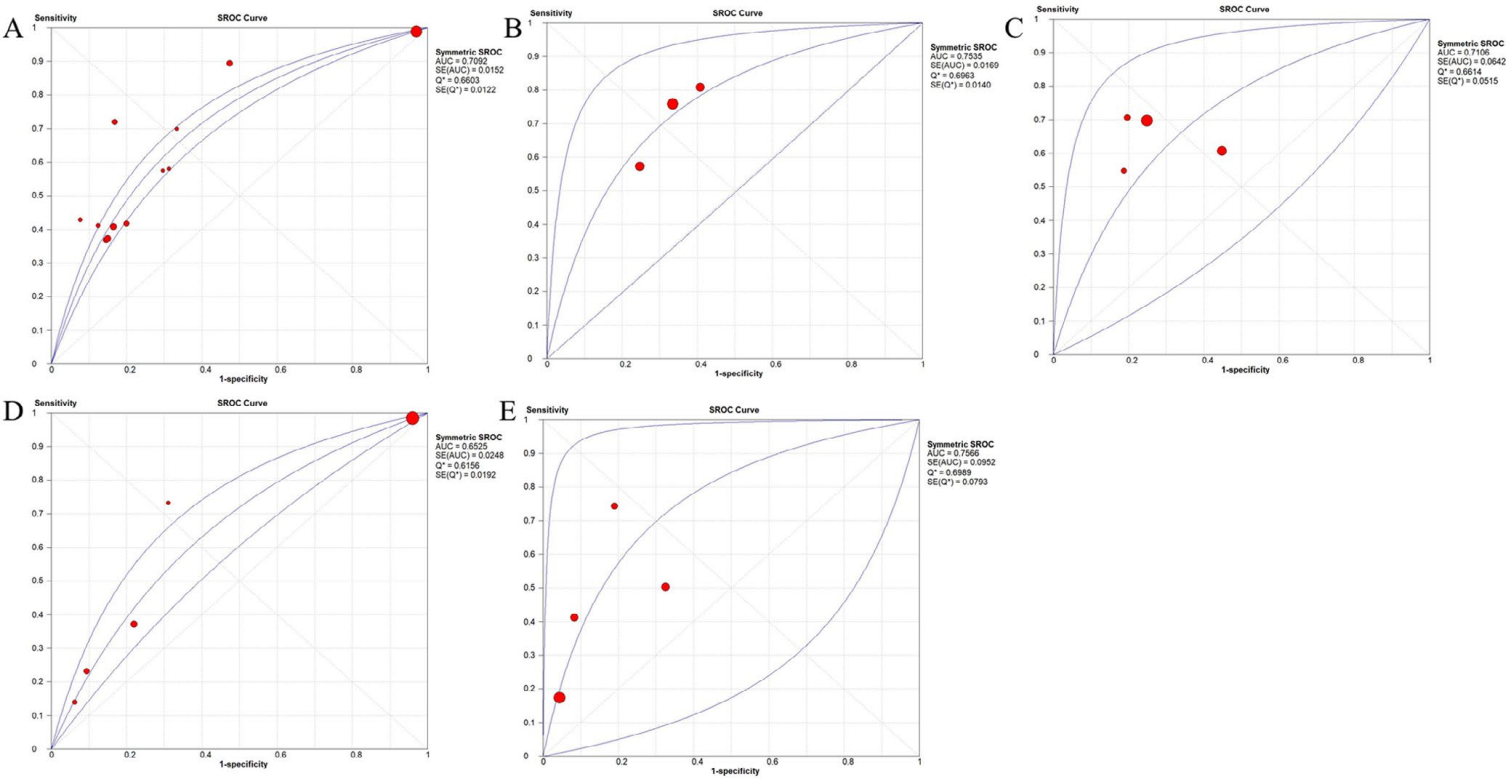
Summary receiver operator characteristic curve to assess the predictive performance of biomarkers. (A) Albumin for IVIG resistance; (B) PNI for IVIG resistance; (C) CAR for IVIG resistance; (D) albumin for CALs; (E) PNI for CALs. AUC, the area under the summary receiver operating characteristic (SROC) curve; CAR, C‐reactive protein to albumin ratio; IVIG, intravenous immunoglobulin; PNI, prognostic nutritional index.

Nine studies with 12 datasets reported the predictive value of ABL levels for IVIG‐resistant KD. The pooled sensitivity was 0.72 (95% CI: 0.70–0.75) with significant heterogeneity (*I* ^2^ = 97.8%; *p* < 0.001); the pooled specificity was 0.38 (95% CI: 0.37–0.39) with significant heterogeneity (*I* ^2^ = 99.8%; *p* < 0.001); the pooled DOR was 3.97 (95% CI: 3.12–5.05) without evidence of heterogeneity (*I* ^2^ = 28.1%; *p* = 0.169). Using the SROC curve, overall AUC was calculated as 0.709 ± 0.015 (Table 5; Figure 4A). Subgroup analyses of studies with retrospective (sensitivity = 0.86; DOR = 5.02; AUC = 0.750 ± 0.027), multicenter (specificity = 0.78; DOR = 4.11; AUC = 0.748 ± 0.025), design and sample size > 300 (sensitivity = 0.74; DOR = 4.19; AUC = 0.727 ± 0.027) were observed to obtain higher predictive potential for ABL than those of the overall results. The heterogeneity for sensitivity was found to be removed in one subgroup stratified by design and sample size, indicating these two factors may be the source of heterogeneity.

**TABLE 5:** Meta‐analysis to assess the predictive performance of nutrition‐associated biomarkers.

Variables					No.	Sensitivity (95% CI), *P*_H_/*I* ^2^	Specificity (95% CI), *P*_H_/*I* ^2^	DOR (95% CI), *P*_H_/*I* ^2^	SROC (AUC ± SE)	Egger
ALB	IVIG resistance	Overall			12	0.72 (0.70–0.75), < 0.001/97.8	0.38 (0.37–0.39), < 0.001/99.8	3.97 (3.12–5.05), 0.169/28.1	0.709 ± 0.015	0.059
		Subgroup	Design	Prospective	4	0.40 (0.35–0.46), 0.176/39.3	0.84 (0.82–0.85), 0.004/77.4	3.23 (2.51–4.18), 0.953/0.0	0.657 ± 0.082	
				Retrospective	8	0.86 (0.83–0.88), < 0.001/97.3	0.26 (0.25–0.27), < 0.001/99.8	5.02 (3.39–7.44), 0.120/38.9	0.750 ± 0.027	
				Multi‐center	11	0.48 (0.44–0.53), < 0.001/83.2	0.78 (0.77–0.80), < 0.001/96.8	4.11 (3.18–5.30), 0.146/31.6	0.748 ± 0.025	
				Single‐center	1	—	—	—	—	
			Sample size	< 300	4	0.59 (0.50–0.68), 0.231/30.3	0.77 (0.71–0.82), 0.003/78.8	3.80 (2.38–6.06), 0.853/0.0	0.704 ± 0.043	
				> 300	8	0.74 (0.71–0.77), < 0.001/98.5	0.37 (0.36–0.38), < 0.001/99.9	4.19 (3.02–5.81), 0.043/51.8	0.727 ± 0.027	
	CALs	Overall			5	0.77 (0.74–0.80), < 0.001/99.2	0.19 (0.18–0.20), < 0.001/99.9	2.56 (1.86–3.50), < 0.669/0.0	0.653 ± 0.025	0.135
		Subgroup	Design	Prospective	1	—	—	—	—	
				Retrospective	4	0.82 (0.79–0.85), < 0.001/99.3	0.11 (0.10–0.12), < 0.001/99.8	2.98 (1.95–4.55), < 0.752/0.0	0.676 ± 0.032	
				Multi‐center	2	0.98 (0.96–0.99), < 0.001/99.3	0.04 (0.04–0.05), < 0.001/98.9	3.25 (1.64–6.45), < 0.298/7.7	—	
				Single‐center	3	0.27 (0.21–0.33), < 0.008/79.5	0.83 (0.80–0.85), < 0.001/93.9	2.38 (1.65–3.42), < 0.714/0.0	0.588 ± 0.084	
			Sample size	< 300	2	0.28 (0.17–0.40), < 0.001/94.7	0.87 (0.80–0.93), 0.001/90.4	3.65 (1.47–9.08), < 0.335/0.0	—	
				> 300	3	0.82 (0.79–0.84), < 0.001/99.5	0.17 (0.16–0.18), < 0.001/99.9	2.43 (1.74–3.41), < 0.681/0.0	0.645 ± 0.027	
PNI	IVIG resistance				3	0.71 (0.66–0.76), 0.001/85.1	0.67 (0.65–0.69), < 0.001/94.6	5.34 (3.98–7.15), 0.300/17.1	0.754 ± 0.017	0.981
	CALs				4	0.43 (0.38–0.48), < 0.001/93.3	0.90 (0.88–0.92), < 0.001/96.5	5.27 (2.40–11.58), 0.001/81.4	0.757 ± 0.095	0.05
CAR	IVIG resistance				4	0.66 (0.61–0.71), 0.255/26.1	0.69 (0.67–0.72), < 0.001/95.4	5.00 (2.26–11.08), < 0.001/85.6	0.711 ± 0.064	0.938

Abbreviations: ALB, albumin; AUC, the area under the summary receiver operating characteristic (SROC) curve; CALs, coronary artery lesions; CAR, C‐reactive protein to albumin ratio; CI, confidence interval; DOR, diagnostic odds ratio; IVIG, intravenous immunoglobulin; *P*_H_, significance for heterogeneity; PNI, prognostic nutritional index; SE, standard error.

#### 3.3.2 PA

Four studies measured the PA levels in IVIG‐resistant and IVIG‐sensitive KD patients. Random‐effect model was used for the pooled analysis because of the presence of significant heterogeneity (*I* ^2^ = 89.3%; *p* < 0.001). The pooled results showed IVIG‐resistant patients had significantly lower PA levels than IVIG‐sensitive ones (SMD = −0.72; 95% CI: −1.17, −0.26; *p* = 0.002) (Table 2).

#### 3.3.3 PNI

**FIGURE 5:**
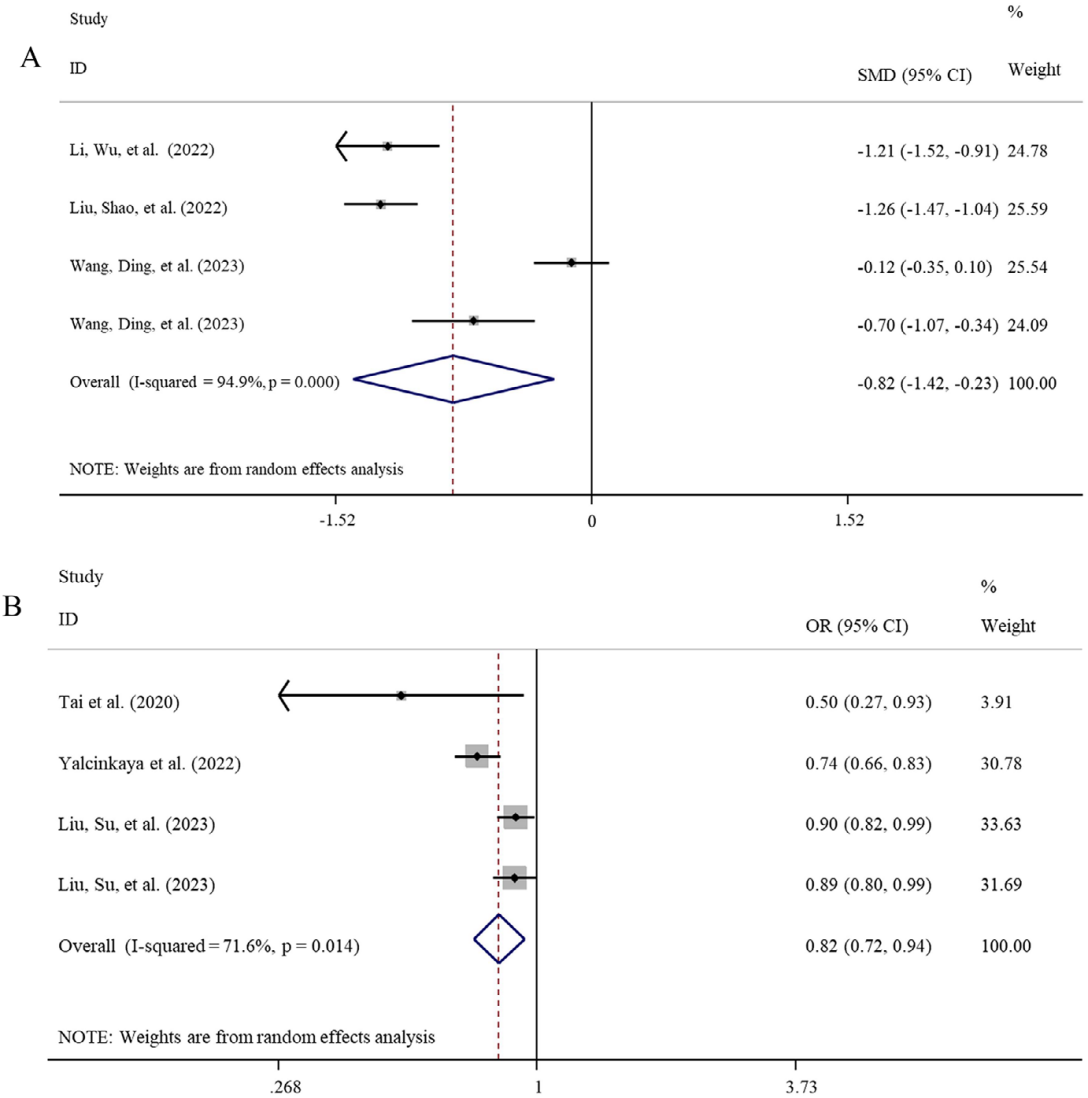
Meta‐analysis to evaluate the associations between PNI and IVIG resistance/CALs in KD patients. (A) PNI difference between IVIG‐resistant and IVIG‐sensitive KD patients in subgroup analysis of studies with sample size > 300; (B) the risk to develop CALs when comparing the high PNI group with the low PNI group. CALs, coronary artery lesions; CIs, confidence intervals; IVIG, intravenous immunoglobulin; KD, Kawasaki disease; OR, odds ratio; PNI, prognostic nutritional index; SMD, standardized mean difference.

Four studies with seven datasets reported the mean PNI in IVIG‐resistant and IVIG‐sensitive KD patients. As there was significant heterogeneity among these studies, a random‐effect model was used (*I* ^2^ = 96.6%, *p* < 0.001). The combined results revealed that there was no significant difference in the PNI between IVIG‐resistant and IVIG‐sensitive patients (SMD = −0.34; 95% CI: −0.96, 0.29; *p* = 0.290) (Table 2). However, the subgroup analysis of studies with sample size > 300 showed the PNI was significantly reduced in IVIG‐resistant patients compared to the IVIG‐sensitive controls (SMD = −0.82, *p* = 0.007) (Table 3; Figure 5A), although the heterogeneity was still present.

Five studies with six datasets explored the association between PNI and the risk of IVIG resistance in KD patients. Under the random‐effect model (*I* ^2^ = 91.5%, *p* < 0.001), results of the meta‐analysis indicated no significant association of PNI with the risk of IVIG resistance in KD patients (OR = 1.04, 95% CI: 0.99–1.09, *p* = 0.149) (Table 2). Subgroup analysis of studies with sample size > 300 observed a low PNI was associated with a higher risk for IVIG resistance, but this association was only at a marginal significance with the random‐effect model analysis (OR = 1.08, *p* = 0.073) (Table 3).

Three studies analyzed the predictive performance of PNI for IVIG resistance. The meta‐analysis concluded that PNI may be a good biomarker for differentiating IVIG‐resistant from IVIG‐sensitive KD patients, with the AUC of 0.754 ± 0.017 (Figure 4B), sensitivity of 0.71 (95% CI: 0.66–0.76), specificity of 0.67 (95% CI: 0.65–0.69), and DOR of 5.34 (95% CI: 3.98–7.15) (Table 5).

#### 3.3.4 BAR

Three studies with six datasets assessed the differences between BAR in IVIG‐resistant and IVIG‐sensitive KD patients. Pooled estimates based on the random‐effect model (*I* ^2^ = 94.1%, *p* < 0.001) showed significantly elevated BAR in KD individuals with IVIG resistance relative to IVIG‐sensitive patients (SMD = 1.10, 95% CI: 0.49–1.72, *p* < 0.001) (Table 2). Subgroup analysis considered this significance may be mainly contributed by studies with sample size < 300 (SMD = 0.84, *p* < 0.001), during analysis of which the heterogeneity was diminished (Table 3).

Three studies assessed the link between BAR and the risk of IVIG resistance in KD patients. Different from the statistical results of SMD, a higher BAR was not found to be significantly associated with the risk of IVIG resistance (OR = 1.99, 95% CI: 0.66–6.02, *p* = 0.226) (Table 2).

#### 3.3.5 CAR

**FIGURE 6:**
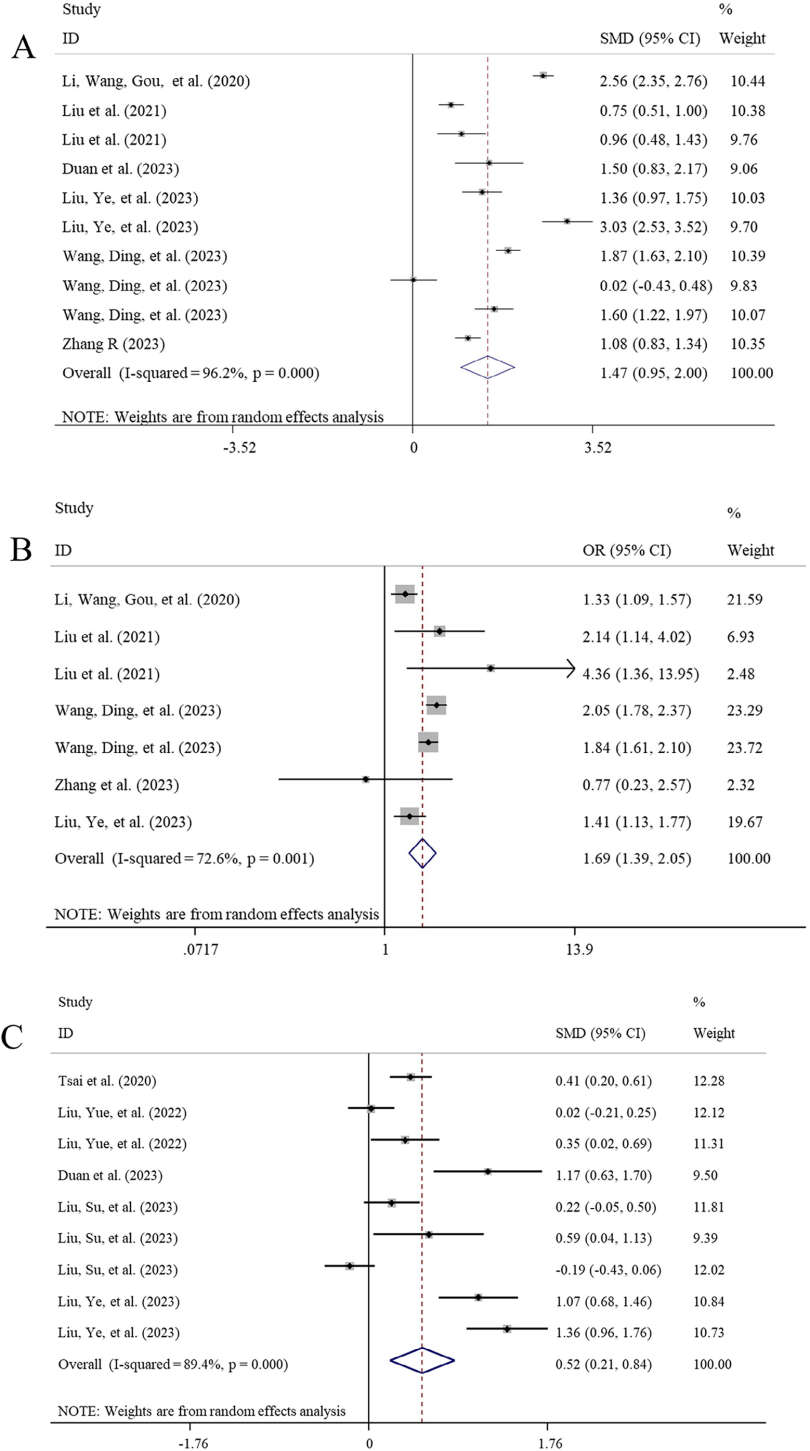
Meta‐analysis to evaluate the associations between CAR and IVIG resistance/CALs in KD patients. (A) CAR difference between IVIG‐resistant and IVIG‐sensitive KD patients; (B) the risk to develop IVIG resistance when comparing the high CAR group with the low CAR group; (C) CAR difference between CAL and non‐CAL KD patients. CALs, coronary artery lesions; CAR, C‐reactive protein to albumin ratio; CIs, confidence intervals; IVIG, intravenous immunoglobulin; KD, Kawasaki disease; OR, odds ratio; SMD, standardized mean difference.

Six studies with 10 datasets investigated the differences of CAR in IVIG‐resistant and IVIG‐sensitive KD patients. Findings from the random‐effect model (*I* ^2^ = 96.2%, *p* < 0.001) showed CAR was significantly higher in KD individuals with IVIG resistance compared to IVIG‐sensitive controls (SMD = 1.47, 95% CI: 0.95–2.00, *p* < 0.001) (Table 2; Figure 6A). This result was still significant in all of subgroups based on design (prospective: SMD = 0.80; retrospective: SMD = 1.63; single‐center: SMD = 1.35; *p* < 0.001), sample size (< 300: SMD = 1.37, *p* = 0.005; > 300: SMD = 1.57, *p* < 0.001), and indicator acquisition time (pre‐IVIG: SMD = 1.31, *p* = 0.003) (Table 3). Study design may be the source of heterogeneity because of a fixed‐effect model used for one subgroup.

Five studies with seven datasets investigated the association of CAR with the risk of IVIG resistance in KD patients. The qualitative synthesis with the random‐effect model (*I* ^2^ = 72.6%, *p* = 0.003) revealed that increases of CAR by one unit resulted in a 1.69‐fold increased risk of IVIG resistance (95% CI: 1.39–2.05, *p* < 0.001; Figure 6B). This result was still significant in most of subgroups based on design (prospective: OR = 2.56, *p* = 0.002; retrospective: OR = 1.62; single‐center: OR = 1.81; *p* < 0.001), sample size (> 300: OR = 1.72, *p* < 0.001), OR source (multivariable: OR = 1.59, *p* < 0.001), and indicator acquisition time (pre‐IVIG: OR = 1.77, *p* < 0.001) (Table 3). Study design was also considered as the source of heterogeneity because of a fixed‐effect model used for prospective subgroup.

The predictive value of CAR for IVIG resistance was assessed in three studies with four datasets. The pooled AUC values (0.711 ± 0.064) found that the CAR had a good discriminatory power to predict IVIG resistance (Figure 4C). Summary sensitivity and specificity were 0.66 (95% CI: 0.61–0.71) and 0.69 (95% CI: 0.67–0.70), with a DOR of 5.00 (95% CI: 2.26–11.08) (Table 5).

### 3.4 Meta‐Analysis to Screen Predictors for CALs of KD Patients

#### 3.4.1 ABL

**FIGURE 7:**
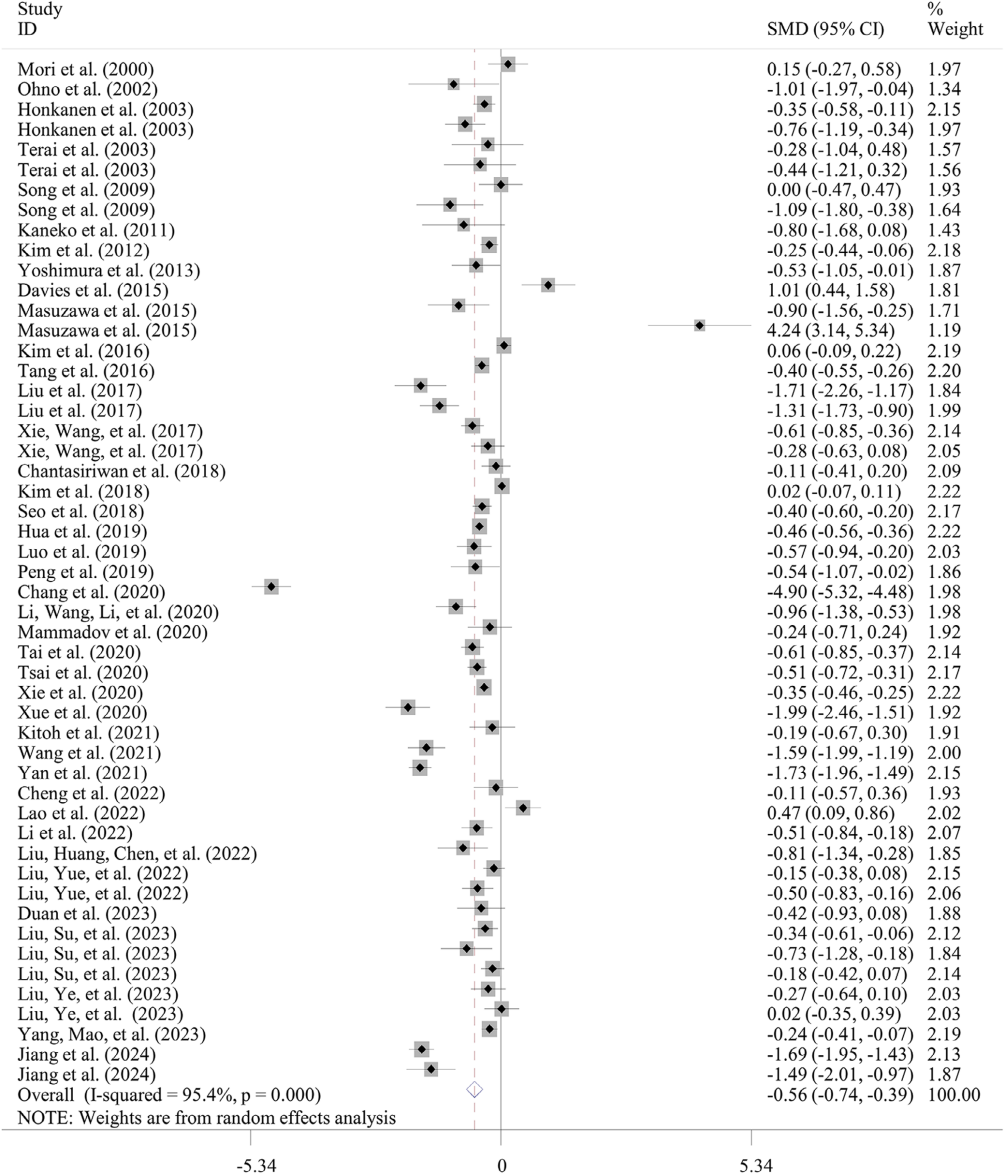
Meta‐analysis of differences in albumin level between CAL and non‐CAL KD patients. CALs, coronary artery lesions; CIs, confidence intervals; KD, Kawasaki disease; SMD, standardized mean difference.

Forty studies with 51 datasets examined the ABL levels in KD patients who developed or did not develop CALs. The meta‐analyses with a random‐effect model (*I* ^2^ = 95.4%, *p* < 0.001) revealed that compared with non‐CAL group, the ABL level was significantly lower in the CAL ones (SMD = −0.56; 95% CI: −0.74, −0.39; *p* < 0.001) (Table 2; Figure 7). Subgroup analysis indicated the ABL level in the CAL ones was particularly lower when it was examined before IVIG therapy (SMD = −0.64, *p* < 0.001), in Asian populations (SMD = −0.59, *p* < 0.001) and in a single‐center trial (SMD = −0.65, *p* < 0.001) (Table 3). The design (prospective: SMD = −0.79; retrospective: SMD = −0.50) or sample size (< 300: SMD = −0.45; > 300: SMD = −0.75) did not influence the overall results, all the analysis results of which were significant (*p* < 0.001) (Table 3). Importantly, significantly lower ABL levels were observed between CAA and controls (SMD = −0.62, *p* < 0.001), but not between CAD and controls (*p* = 0.164) (Table 3). The heterogeneity was still present for these subgroup analyses, and thus, the causes of heterogeneity were unknown, which was demonstrated in meta‐regression analysis (Table 4).

**FIGURE 8:**
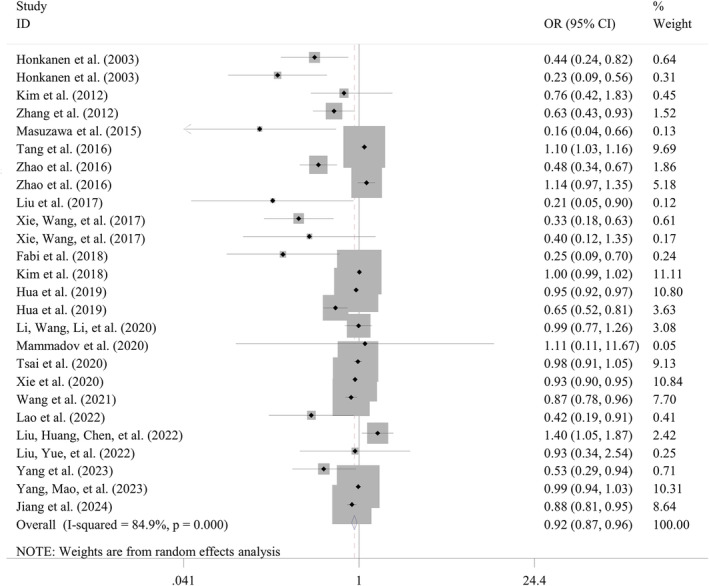
Meta‐analysis of the risk to develop CALs in KD patients when comparing the high albumin level group with the low albumin level group. CALs, coronary artery lesions; CIs, confidence intervals; KD, Kawasaki disease; OR, odds ratio.

Twenty‐two studies with 26 datasets reported the association of ABL levels and the risk of CALs in KD patients. Under the random‐effect model (*I* ^2^ = 85.5%; *p* < 0.001), the pooled analysis demonstrated that a high ABL level was associated with a decreased risk to develop CALs (OR = 0.92, 95% CI: 0.87–0.96, *p* = 0.001) (Table 2; Figure 8). Subgroup analysis found this significant association existed in experiments with a single‐center (OR = 0.90, *p* = 0.006), retrospective (OR = 0.93, *p* = 0.003) study design, but not for prospective and multicenter trials (*p* > 0.05). Significant results were revealed in both subgroups stratified by country (Asian: OR = 0.93, *p* = 0.004; non‐Asian: OR = 0.33, *p* < 0.001), sample size (< 300: OR = 0.64, *p* = 0.006; > 300: OR = 0.94, *p* = 0.008), and OR source (univariable: OR = 0.63, *p* = 0.022; multivariable: OR = 0.92, *p* = 0.002) (Table 3). Similarly, lower ABL levels were associated with an increased incidence of CAA (OR = 0.60, *p* = 0.018) (Table 3). The heterogeneity was eliminated in analysis of non‐Asian populations, suggesting the ethnicity may be the potential source of heterogeneity. Meta‐regression analysis indicated study design, sample size, and CAL type were all contributors for the heterogeneity (Table 4).

Four studies with five datasets reported the fair power of ABL levels in predicting CALs in KD patients. The pooled outcomes were as follows: sensitivity, 0.77 (95% CI: 0.74–0.8), specificity, 0.19 (95% CI: 0.18–0.20); DOR, 2.56 (95% CI: 1.86–3.50), and AUC, 0.653 ± 0.025 (Table 5; Figure 4D). The sensitivity (0.82), DOR (2.98), and AUC (0.676 ± 0.032) were enhanced in subgroup analyses of four studies with retrospective design (Table 5). Also, no heterogeneity was present for analysis of DOR in overall and subgroup analyses, implying the results were reliable.

#### 3.4.2 PNI

Four studies with seven datasets provided the PNI data of CALs and non‐CAL KD patients. As there was significant heterogeneity among these studies, a random‐effects model was used (*I* ^2^ = 61.9%, *p* = 0.015). The meta‐analysis result revealed that there was no significant difference in the PNI between CALs and non‐CAL patients (SMD = −0.20; 95% CI: −0.40, 0.01; *p* = 0.052) (Table 2). However, subgroup analysis showed compared with controls, the PNI was only lower in CAA patients than that in controls (SMD = −0.18, *p* = 0.013) (Table 3). Also, a fixed‐effect model was used for analysis of CAA, indicating CAL type may be the potential source of the heterogeneity.

Three studies with four datasets evaluated the association between PNI and the risk to develop CALs in KD patients. Under the random‐effect model (*I* ^2^ = 71.6%, *p* = 0.014), the meta‐analysis indicated high PNI was significantly associated with a decreased risk to develop CALs in KD patients (OR = 0.82, 95% CI: 0.72–0.94, *p* = 0.003) (Table 2; Figure 5B).

Four studies explored the predictive ability of PNI in determining coronary involvement among patients with KD. The meta‐analysis concluded that PNI may be a good predictor for identifying the KD patients with the high risk to develop CALs, with the AUC of 0.757 ± 0.095 (Figure 4E), sensitivity of 0.43 (95% CI: 0.38–0.48), specificity of 0.90 (95% CI: 0.88–0.92), and DOR of 5.27 (95% CI: 2.40–11.58) (Table 5).

#### 3.4.3 BAR

Two studies with three datasets assessed the differences between BAR in CALs and non‐CAL KD patients. Pooled estimates based on a fixed‐effect model (*I* ^2^ = 40.4%, *p* = 0.258) showed no difference between BAR in KD patients with CALs compared to controls (SMD = 0.13; 95% CI: −0.11, 0.36; *p* = 0.285) (Table 2).

#### 3.4.4 CAR

Five studies with nine datasets investigated the differences of CAR in CALs and non‐CAL KD patients. Findings from the random‐effect model (*I* ^2^ = 89.4%, *p* < 0.001) showed there were higher levels of CAR in KD patients with CALs compared to non‐CAL controls (SMD = 0.52, 95% CI: 0.21–0.84, *p* = 0.001) (Table 2; Figure 6C). Subgroup analysis also found CAR was particularly higher in CAA subtype of CALs (SMD = 0.59, *p* = 0.006) (Table 3). Analysis of sample size < 300 (SMD = 0.56, *p* = 0.011) and pre‐IVIG CAR (SMD = 0.41, *p* = 0.005) also demonstrated increased CAR levels in KD patients with CALs relative to non‐CAL controls. The source of the heterogeneity may be indefinite because the heterogeneity was not removed in all subgroups and negative results in meta‐regression analysis (Table 4).

#### 3.4.5 AGR

Three studies with six datasets compared AGR in CALs and non‐CAL KD patients. Findings from the random‐effect model (*I* ^2^ = 56.7%, *p* = 0.042) showed the difference of AGR between CALs and non‐CAL KD patients was not statistically significant (SMD = −0.19; 95% CI: −0.39, 0.01; *p* = 0.066) (Table 2). Subgroup analysis also did not detect a decrease of AGR in CALs relative to non‐CAL KD patients (Table 3), although the heterogeneity was excluded after stratification by sample size.

Pooling the OR data of three studies with four datasets also found no significant association of AGR and the risk to develop CALs (OR = 0.82, 95% CI: 0.34–1.96, *p* = 0.655) under a fixed‐effect model (*I* ^2^ = 0%, *p* = 0.883) (Table 2).

### 3.5 Publication Bias and Sensitivity Analysis

**FIGURE 9:**
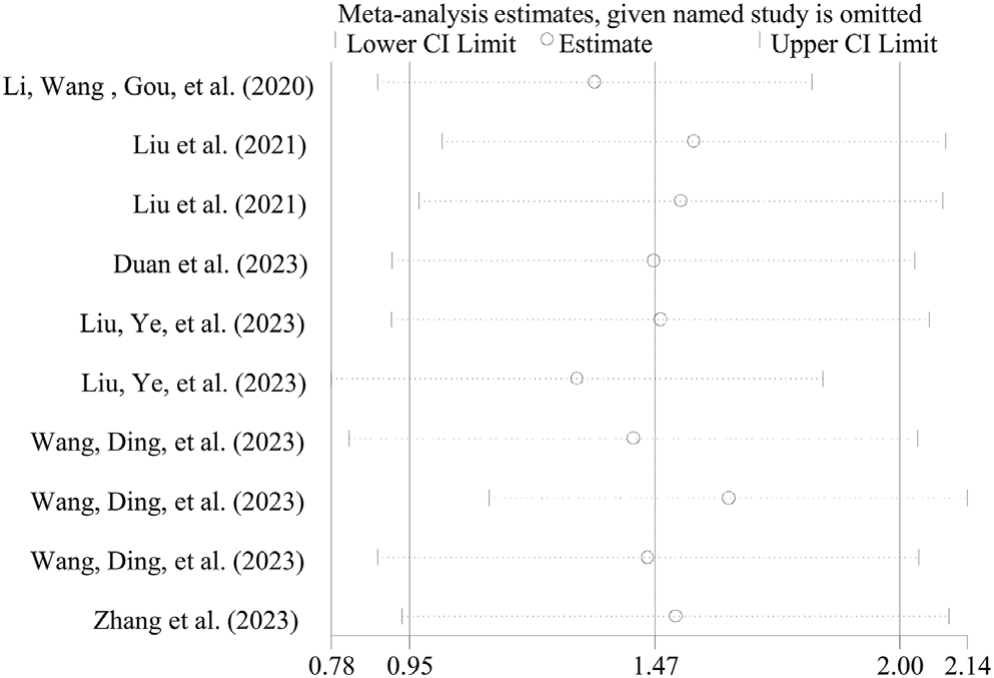
A representative sensitivity analysis result for CAR in predicting IVIG resistance. CAR, C‐reactive protein to albumin ratio; CI, confidence interval; IVIG, intravenous immunoglobulin.

Egger's linear regression test showed the presence of publication bias to use the ABL for predicting IVIG resistance (*p* < 0.001) and CALs (*p* < 0.001), to use the BAR to predict IVIG resistance (*p* = 0.024), to use CAR to predict CALs (*p* = 0.047) (Table 2), as well as to use PNI to predict CALs (Table 5). No evidence of publication bias was present for other biomarkers (Table 2). The trim‐and‐fill method was then used to adjust the effects of publication bias for these biomarkers. However, the results were still not changed relative to pre‐correction except for CAR to predict CALs (*p* = 0.358). Sensitivity analysis also confirmed the stability of the meta‐analysis results (Figure 9).

## 4 Discussion

Compared with the studies of Chen et al. (2011) (51 vs. 10); Baek and Song (2016) (76 vs. 12) and Liu, Wang, and Du (2020) (76 vs. 19), our updated meta‐analysis included more articles or datasets to assess the ALB levels in KD patients with CALs or IVIG resistance; thus, our conclusion may be more convincing (although all of them confirmed ALB level was significantly lower in patients with CALs or IVIG resistance relative to controls). In addition to the mean ALB levels, the OR values that predicted the risk to develop CALs or IVIG resistance were, for the first time, extracted from 40 and 26 datasets and pooled. In line with the pooled SMD, we also found a higher ALB level significantly predicted the lower risk to develop CALs or IVIG resistance in KD patients. To further demonstrate the predictive value of ALB, our study pooled the four‐table data from individual studies (12 or 5), which were not investigated previously. The meta‐analysis results showed the overall sensitivity (AUC of SROC) of ALB for diagnosis of IVIG resistance and CALs can reach 0.72 (0.709) and 0.77 (0.653), respectively, but the specificity seemed to be relatively inferior (< 0.5). Recently, there have some articles to assess the association of ALB‐related combined biomarkers with IVIG resistance and CALs in KD patients, but no study used the meta‐analysis to provide reliable evidence. By synthetizing the published articles, our overall and/or subgroup analyses verified differences in the mean PNI and CAR levels were statistically significant between IVIG‐resistant (CALs) and IVIG‐sensitive (non‐CALs) patients; high PNI or lower CAR was associated with the decreased risk to develop IVIG resistance and CALs. As expected, the sensitivity, specificity, and AUCs of SROC of CAR and PNI for diagnosis of IVIG resistance were all > 0.6; the specificity was 0.9 and AUC of SROC of PNI were 0.757 for diagnosis of CALs. Furthermore, PA and BAR were observed to be significantly different in IVIG‐resistant patients compared with IVIG‐sensitive controls, but their predictive performance was not reported in included studies. AGR was only compared between CAL and non‐CAL patients in included studies, but the pooled analysis did not examine the difference and the risk association (*p* > 0.05).

The underlying mechanism of hypoalbuminemia in KD children with poor outcomes remained unclear, but it was speculated to be attributed to the following reasons: (1) pathologically, KD is a systemic vasculitis, while the liver has a rich vasculature. Thus, hepatic dysfunction is a common complication during acute KD episodes (Mammadov et al. 2020). In the condition of inflammation that could not be down‐regulated by high‐dose IVIG (Lee et al. 2005), CRP was persistently produced (Okada et al. 2015; Tomita et al. 2019). The production of acute phase protein CRP by the hepatocytes may compromise the synthesis of PA and ALB, showing significant negative correlations (Mammadov et al. 2020); (2) a lack of essential amino acids (such as arginine, asparagine, isoleucine, leucine, lysine, methionine, phenylalanine, proline, threonine, and tryptophan) due to low nutrient intake or malnutrition reduced albumin synthesis rates (Arques 2018a; Kelman et al. 1972); (3) in vitro studies showed the proinflammatory factors (transforming growth factor‐beta1 and tumor necrosis factor‐alpha [TNF‐α]) can up‐regulate vascular endothelial growth factor and contribute to hyper‐permeability of local blood vessels in KD patients (Huang and Zhang 2021; Terai et al. 1999), which ultimately led to increased leakage of PA and ALB.

Several studies reported that ALB could exert anti‐inflammatory functions (Arques 2018b). For example, Powers et al. (2003) found resuscitation with 25% ALB significantly reduced the degree of histopathological injury of lung by diminishing bronchoalveolar lavage fluid neutrophil counts, cytokine‐induced neutrophil chemoattractant messenger RNA concentrations, and nuclear factor‐kappaB translocation compared with resuscitation with Ringer's lactate. By in vivo (mice), ex vivo (precision‐cut liver slices), and in vitro (primary hepatocytes) studies, Duran‐Güell et al. (2021) found administration of human ALB protected against TNFα‐induced liver injury. Xie, Guo, et al. (2017) identified ALB could significantly mitigate early neurovascular dysfunction of subarachnoid hemorrhage rats through suppression of the polarization of macrophages/microglia to the M1 phenotype (interleukin‐1β, CD16, and CD32) and neutrophil invasion (decreased mRNA levels of monocyte chemoattractant protein‐1, cytokine‐induced neutrophil chemoattractant‐1, and CXC chemokine ligand 2). Thus, the downregulation of ALB may further strengthen the roles of pro‐inflammatory mediators and result in the progression of KD and the damages of coronary arteries (Sawashita et al. 2023). Furthermore, ALB resuscitation was reported to improve lipopolysaccharide‐induced fractional shortening of cardiomyocytes by reducing the messenger RNA expression of an oxidant mediator nitric oxide synthase II (Walley et al. 2003), which indicated oxidant stress induced due to ALB loss may be another mechanism for explaining IVIG resistance and the development of CALs.

In addition to ALB, the combined biomarker PNI brings the indicator of lymphocytes. In acute Epstein–Barr virus (EBV)‐DNA (−) infected and latent EBV‐infected KD patients, the incidence of CALs was found to be significantly higher, which was accompanied by decreased number of CD8^+^ T lymphocytes and downregulation of anti‐inflammatory molecule α7 nicotinic acetylcholine receptor released by CD8^+^ T lymphocytes (Tao et al. 2023). Several investigators also detected that lymphocyte counts were significantly decreased in IVIG‐resistant KD patients (Takeshita et al. 2017; Tan et al. 2019). Thus, low PNI was associated with a high risk of CALs and IVIG resistance, which was confirmed in our meta‐analysis. CRP has been recognized to reflect the severity of systemic and local inflammation. Several meta‐analyses had confirmed the higher CRP was associated with IVIG resistance (Baek and Song 2016; Liu and Wu 2022; Liu, Wang, and Du 2020) and CALs (Yan et al. 2019) in KD patients. CRP was revealed to support the development and progression of CALs via stimulating the expression of receptor for advanced glycation end products and promoting the release of circulating endothelial cells from human coronary endothelial cells (Zhou et al. 2015). The high combined biomarker CAR may represent the presence of aggressive inflammatory response due to an imbalance between pro‐inflammatory and anti‐inflammatory mediators. Therefore, high CAR predicted a high risk to develop CALs and IVIG resistance, which was also verified in our meta‐analysis. Similar to hypoalbuminemia, hyperbilirubinemia is another result from the damages of hepatocytes, which prevents the conversion of bilirubin into bile or cholestasis (Cheng et al. 2020). Theoretically, hypoalbuminemia and hyperbilirubinemia as well as high BAR were contributors for IVIG resistance and CALs of KD patients. The association of first two indicators with IVIG resistance were demonstrated in the previous studies (Baek and Song 2016; Liu, Wang, and Du 2020) and the association of BAR with IVIG resistance was detected in our study. However, BAR was not found to predict the risk to develop CALs, which may be attributed to weak harmful roles of hyperbilirubinemia (Yan et al. 2019). Even, there was evidence to indicate elevated serum bilirubin concentrations protected from coronary micro‐vascular dysfunction via playing anti‐inflammatory effects (Gullu et al. 2005; Yoshino et al. 2011). Future studies are hereby needed to confirm the relationship between BAR and CALs in KD patients.

Several different risk scoring models had been established by Japanese teams according to multiple indicators to predict IVIG resistance and CALs in KD patients, including Harada (white blood cells, platelet, CRP, hematocrit, ALB, age, and sex) (Harada 1991), Kobayashi (day of illness at initial treatment, age, white blood cells, platelet, aspartate aminotransferase, sodium, and CRP) (Kobayashi et al. 2006), Egami (Egami et al. 2006), and Sato (CRP, total bilirubin, and aspartate aminotransferase) (Sano et al. 2007). However, the sensitivity or specificity of these scoring models seemed to be significantly decreased (< 0.5) when using the patients from other countries, such as Korea and China (Fu, Du, and Pan 2013; Qian et al. 2018; Tan et al. 2019; Wang, Huang, et al. 2023). These findings indicated the requirement to develop the model with higher predictive capacity and applicable to all countries. From the results of AUC of SROC, we found the combined biomarkers were more effective to predict IVIG resistance and CALs in KD patients. Thus, we considered combined biomarkers (PNI, CAR) should be commonly used with ALB to construct a new risk scoring. This hypothesis can be indirectly proved by the study of Wang, Ding, et al. (2023) (lymphocytes, aspartate aminotransferase, sodium, total bilirubin, and CAR), in which the AUC was 0.825 to predict IVIG resistance (> 0.709 for ALB); as well as the study of Wang et al. (2024), in which the AUC of lymphocyte‐CRP‐ALB was reported to reach 0.781 (> 0.653 for ALB), in predicting CALs in KD patients.

The present study had some limitations. First, the number of included studies for analysis of ALB‐combined biomarkers was relatively small and most of them (even all) explored Asian patients (because the incidence of KD is 10–20 times higher in Asian than European and American countries; thus, it may be difficult to collect adequate samples on non‐Asian children, which results in less related studies published) (Rowley and Shulman 2018). Thus, whether these combined biomarkers (like ABL) were associated with IVIG resistance and CALs in non‐Asian KD patients needed further confirmation. Second, most of included studies were in a retrospective and single‐center design, which may introduce unavoidable bias in patient selection and data collection and limit data homogeneity. Third, there was substantial heterogeneity for analysis of several indicators (e.g., ALB), and the causes of heterogeneity could not be confirmed after the subgroup or meta‐regression analyses. Fourth, the threshold value was not reported or unified in different articles, which influenced its generalization in clinical practice. Fifth, in addition to CALs, whether consequential cardiovascular complications (myocarditis, valvular regurgitations, pericarditis, KD shock syndrome, and MACE) were significantly associated with these biomarkers remained undetermined because of rare reports (Liu, Shao, et al. 2022; Rigante et al. 2024). Sixth, other ALB‐merged biomarkers (such as neutrophil percentage‐to‐ALB ratio) should be further explored to screen the optimal predictor for KD patients (Deng et al. 2024). Accordingly, more studies with a prospective, multiple‐center design, larger sample size, and KD patients from other ethnicities are needed to further confirm the predictive values of ALB and ALB‐related combined biomarkers for IVIG resistance, CALs, and cardiovascular sequelae in the future.

## 5 Conclusion

In the present study, we comprehensively assessed the association of ALB and ALB‐combined biomarkers with IVIG resistance and CALs of KD children by performing a meta‐analysis of 94 published clinical articles. Regardless of analyzing SMD or OR data, the pooled results showed lower ALB and higher CAR were associated with increased risks of IVIG resistance and CALs (particularly CAA) in KD patients. The synthetic analysis of diagnostic studies indicated the AUCs of ALB and CAR in predicting IVIG resistance and CALs reached 0.709, 0.653, and 0.711, respectively. Furthermore, pooling the SMD data from more than 300 samples and OR data, respectively, demonstrated PNI were reduced in IVIG‐resistant KD patients and high PNI predicted a decreased risk of CALs (particularly CAA). The predictive potential of PNI for IVIG resistance and CALs achieved 0.754 and 0.757, respectively. The mean level of PA was also found to be lowed, while BAR was elevated in IVIG‐resistant patients compared with controls. These findings suggest ALB, CAR, and PNI may serve as promising biomarkers for stratification of CAL or IVIG resistance patients. PA and BAR can be added to the prediction system for IVIG resistance. These biomarkers may benefit the clinical decision‐making upon early initiation of intensified therapy to improve prognostic outcomes. However, the current evidence is limited, and more studies are needed to assess their predictive accuracy and generalizability in the future.

## Author Contributions

**Ling Liu:** conceptualization (equal), data curation (equal), formal analysis (equal), writing – original draft (equal). **Rui Chen:** data curation (equal), investigation (equal), writing – original draft (supporting). **Hong Wang:** investigation (equal), methodology (equal), writing – original draft (supporting). **Honglu Yu:** resources (equal), software (equal), writing – original draft (supporting). **Zeyu Ai:** methodology (equal), visualization (equal), writing – original draft (supporting). **Xiaofei Zhang:** conceptualization (equal), supervision (equal), writing – review and editing (equal).

## Conflicts of Interest

The authors declare no conflicts of interest.

## Data Availability

The original contributions presented in this study are included in the article, further inquiries can be directed to the corresponding author.
